# Joint Stochastic Spline and Autoregressive Identification Aiming Order Reduction Based on Noisy Sensor Data

**DOI:** 10.3390/s20185038

**Published:** 2020-09-04

**Authors:** Dan Stefanoiu, Janetta Culita

**Affiliations:** Faculty of Automatic Control and Computers, Politehnica University of Bucharest, 060042 Bucharest, Romania; dan.stefanoiu@upb.ro

**Keywords:** stochastic cubic spline, ARMAX identification models, hill climbing, metaheuristics

## Abstract

This article introduces the spline approximation concept, in the context of system identification, aiming to obtain useful autoregressive models of reduced order. Models with a small number of poles are extremely useful in real time control applications, since the corresponding regulators are easier to design and implement. The main goal here is to compare the identification models complexity when using two types of experimental data: raw (affected by noises mainly produced by sensors) and smoothed. The smoothing of raw data is performed through a least squares optimal stochastic cubic spline model. The consecutive data points necessary to build each polynomial of spline model are adaptively selected, depending on the raw data behavior. In order to estimate the best identification model (of ARMAX class), two optimization strategies are considered: a two-step one (which provides first an optimal useful model and then an optimal noise model) and a global one (which builds the optimal useful and noise models at once). The criteria to optimize rely on the signal-to-noise ratio, estimated both for identification and validation data. Since the optimization criteria usually are irregular in nature, a metaheuristic (namely the advanced hill climbing algorithm) is employed to search for the model optimal structure. The case study described in the end of the article is concerned with a real plant with nonlinear behavior, which provides noisy acquired data. The simulation results prove that, when using smoothed data, the optimal useful models have significantly less poles than when using raw data, which justifies building cubic spline approximation models prior to autoregressive identification.

## 1. Introduction

Over the last few decades, the spline functions theory has been applied in many fields of science and engineering, such as: signal processing [1], computer graphics [2], system modeling [3] and identification [4], statistics [5], industrial design [6], geodetics [7], etc. In parallel, substantial effort has been made towards developing the mathematical bases of spline functions [8]. As already known, the spline function is a piecewise polynomial continuous curve passing through some pre-defined points, referred to as *joint/control points* or *knots*. Usually, such a knot consists of a time instant and a data value. Nevertheless, a knot can be a spatial location as well, together with its coordinates [7,9]. 

Two major approaches are commonly reported into the scientific literature, for deriving a spline function [10]: (a) by enforcing the polynomials to pass through successive knots in a table, subjected to some continuity and, possibly, derivative constraints at their knots; (b) by simultaneously determining the polynomials and their knots, while optimizing some cost function, subject to similar constraints, as previously described. In the first case, the spline function is a data interpolator and can be determined by algebraic methods. In the second case, the spline function is a data approximation model or a curve fitting model and can be determined by variational methods [10,11]. Usually, the curve fitting is realized by means of *Least Squares* (LS) method [7,12,13,14]. The data smoothing effect is obtained especially when using third degree polynomials, which involves enforcing continuity constraints up to the first derivative. The smoothness is an important feature, usually exploited in spline approximation, which depends on the closeness to the data points. Thus, a spline model closer to each data point can be less smooth than a spline model closer to a (sub)set of data points, in the LS sense.

Within this article, one investigates how the spline approximation concept can be employed in *System Identification* (SI) [12,13], in order to estimate an optimal useful model with reduced complexity (in terms of poles number). This, in turn, would lead to less complex regulators, which are faster and easier to implement, even in real time environment. However, the control problem is not addressed here. 

Consequently, firstly, one aims to build an optimal and adaptive smooth cubic spline model which best passes near the raw data in the LS sense, while preserving the continuity up to the first derivative. Secondly, one uses the spline model to generate a smoothed data set in view of multivariable process identification. One can prove that the smoothed data lead to a less complex identification model than the raw data. The identification models belong here to ARMAX class, which can be found in a number of identification and control applications (e.g., [12,13,15,16,17]). 

As already known, raw data are affected by stochastic noises (mainly produced by sensors) that tremendously determine both the shape of the final fitted curve and the knots of component polynomials. Under these circumstances, the cubic spline model inherits the stochastic nature of data. 

The main goal of this article is to prove that, by smoothing the raw data with the optimal stochastic spline model, one obtains an optimal useful identification model with fewer poles than when unprocessed, raw data are employed. In order to best estimate the cubic polynomials of spline model, the data subsets are adaptively tailored, by using the error standard deviation of the error between the subset raw data and the simulated identified polynomial. Thus, the number of knots stops to increase when the error standard deviation firmly increases (e.g., has five successive increases). After the data subsets are set, the spline model is built with the help of *Least Squares Method* (LSM) [12]. This approach is non unique. Some other approaches in selecting data segments are reported into the literature and they rely on: fixed number of knots (previously specified) [18], subjective selection [8] or predicted selection with the help of a neural network [19]. 

The problem of smoothing data appeared over the years in many contributions within SI field, but they often exhibit limitations or are too specific to some applications [20]. An early work is [21], where a smooth cubic spline model is derived from minimizing the sum of squares of deviations for each data segment with first derivative continuity constraints, but the segment lengths are fixed (non adaptively set). Moreover, in the simulations, the employed data are extremely smooth, so that a deterministic spline interpolator could have worked as well. In [8], where the problem of estimating the trend of time series is addressed by curve fitting, there is only one free parameter (namely the *smoothing parameter*) chosen by the user, in a subjective manner, which can control the trade-off between the smoothness of the spline model and its closeness to the data. In addition, no optimality criterion is used and, apparently, the procedure takes into account groups of maximum five successive data points. The resulted model is neither optimal nor adaptive. In [9], a cubic B-spline curve approximation is used to predict the missing data in a distributed parameter system, with an unknown structure and parameters. Apparently, the separation between the cubic polynomials does not vary adaptively. Moreover, the condition of unitary sum for the connected piecewise polynomials in each point seems unnatural. In [1], orthogonal polynomial based approximation is introduced, but the case study refers to signals with abrupt changes; without this feature, the data segments corresponding to each polynomial are difficult to set. The article [18] is concerned with nonlinearity identification of a complex dynamic system, by using uniformly sampled knots and a nonlinear basis of functions, which have to be appropriately selected. In [22], the identification of impulse or frequency response by using modified B-splines is investigated, but the choice of the basis functions to build the spline curve is not clearly specified and the case study rather is based on toy examples. In [11], a LS adjustment is adopted for spline approximation and the knots are equidistantly rearranged by an iterative process until some quality criterion is met. Still, the data selection cannot be considered adaptive. In [20] a model-based approach for optimally detecting outliers in GPS trajectories is proposed. To this aim, a cubic smooth spline is firstly estimated to adaptively extract the trend of the GPS observed trajectory. Then, the resulted residuals are used to determine potential outliers by using a time series model (of ARMA type). The spline model ensures an optimal trade-off between the fitness to the data (in LS sense) and certain smoothing requirements (minimizing the energy of the second derivative) determined by an adaptive smoothing parameter. This parameter is obtained by maximizing a criterion that combines the generalized cross-validation and the Akaike information criterion applied on a data sequence, which adapts itself each time an outlier is removed from the current data set.

Although the idea of LS approximation by splines is widely practiced in curve fitting [23], to the best of our knowledge, the impact of an adaptive stochastic spline upon model complexity reduction in multivariable system identification has not been yet investigated within time domain models. 

In order to estimate the optimal identification models, two major approaches are proposed next. Within the first one, a two-step optimization is adopted, where the optimal useful model is separately identified from the model of corrupting perturbation. Within the second one, a global (one-step) optimization is performed, which results in an overall model. Both approaches rely on the *Signal-to-Noise Ratio* (SNR) as maximization criterion for both the identification and validation data sets. Since the SNR usually exhibits a fractal shape (in context of data acquired from physical processes), the optimal structure of each model is determined by means of a metaheuristic. In this respect, the paper introduces an advanced version of the classical *Hill Climbing Algorithm* (HCA), by using groups of climbers [24,25]. Although, nowadays, there is a trend in SI to adhere to non-conventional models (e.g., Long Short Term Memory (LSTM) in [26]), the performed simulations prove that the classical ARMAX model returns good results as well, especially in the context of a spline model. 

The paper is organized as follows. Section 2 firstly offers a detailed presentation of the stochastic cubic spline model, including the design of the smoothing algorithm. Within this section, the problem of multivariable system identification is formulated and solved, by taking into account experimental data. The *Advanced Hill Climbing Algorithm* (AHCA) is described at length in the end. Section 3 describes a case study based on a non-linear multivariable process (a two-tank water installation) and performs a comparative analysis of the simulation results. Some concluding remarks are drawn in Section 4.

## 2. Methods

### 2.1. Building the Adaptive Stochastic Cubic Spline Model

#### 2.1.1. Spline Model Design Principle

Consider a stochastic process that can provide sets of *Input/Output* (I/O) raw acquired data with relatively small SNR (i.e., corrupted by quite strong noises), still acceptable in SI. The global identification model is assumed to be linear and relies on two additive filters: one providing the useful signal from the process inputs and another one generating the colored noise from the white noise. One aims to integrate this model into a process control application. Therefore, it is suitable that the useful filter yields a smooth simulated signal with a reduced number of poles. In other words, one aims to separate as accurately as possible the useful information from the noise information encoded by the I/O data, while employing a small number of useful poles. In order to design such a useful filter, one can define an optimization criterion, which expresses (directly or indirectly) the fitness between the useful signal and the *smoothed* output data. Obviously, this criterion has to be maximized. The filters design is described in the next subsection, where the identification of the global model is extensively discussed. Hereafter, the discussion focuses on how the raw output data can be *smoothed* by using the optimal stochastic spline model.

In order to smooth the raw output data, a stochastic cubic spline model based on adaptive length data subsets is built. This model best approximates all the measured points in the LS sense, passing as close as possible between them. This means the model does not act as spline interpolator of raw data, but as stochastic spline curve fitter. 

The model is built by concatenating the piecewise estimated cubic polynomials that best approximate the successive adaptive length data subsets. Each data subset is not a priori known and depends on how the *standard deviation* (std) of the error between the current polynomial and the corresponding raw data varies. More specifically, this model is estimated for a larger and larger data subset, starting from a minimum number of samples (e.g., 10), until the std begins to firmly increase. To detect such a behavior of std, one accounts a threshold number of successive increases, which can be chosen by the user (e.g., 5). The data subset is enhanced with new values and new knots are created by an averaging technique, as explained in this subsection. Each subset enhancement is followed by re-estimation of cubic polynomial and std. When the number of successive std increases reaches the threshold, the data subset construction is stopped and, thus, the current cubic polynomial is already estimated. Afterwards, a new data subset has to be configured, starting from the last measured data of the previous subset, in order to estimate the next cubic polynomial, and so on.

#### 2.1.2. Spline Model Identification

Denote by $D_{N}={\left\{y[n]\right\}}_{n\in \bar{1,N}}$ the output acquired signal, and by ${\hat{D}}_{N}={\left\{\hat{y}[n]\right\}}_{n\in \bar{1,N}}$ the corresponding simulated signal on the measure horizon, whose length, $N\in {\mathbb{N}}^{*}$, is large enough (at least 100). One assumes that the smoothness of the stochastic spline model is locally analyzed, within the k-th data subset with variable length $M_{k}\in {\mathbb{N}}^{*}$, where $M_{k}\ll N$ and $k\in {\mathbb{N}}^{*}$. As mentioned before, all lengths $M_{k}$ depend on the std increase threshold, being updated simultaneously with the cubic polynomial estimation. For now, assume that all $M_{k}$ are known. Before analyzing the two cases, recall how to estimate the parameters of a linear regression model, which best approximates the measured output data $D_{N}$ in the LS sense. By expressing the regression form:

(1) $$y[n]=\varphi_{}^{T}[n]\theta +e[n],\;\forall n\in \bar{1,N}$$

where $\varphi^{T}[n]$ is the (transposed) regression vector, θ is the vector of unknown parameters and e is a white noise corrupting the data, one can define the optimization criterion below:

(2) $$V\left(\theta \right)=\sum_{n=1}^{N}e^{2}[n]=\sum_{n=1}^{N}{\left(y[n]-\varphi_{}^{T}[n]\theta \right)}^{2}.$$

By minimizing V, the LS estimation is obtained:

(3) $$\hat{\theta }={\left[\sum_{n=1}^{N}\varphi [n]\varphi_{}^{T}[n]\right]}^{-1}\left[\sum_{n=1}^{N}\varphi [n]y[n]\right]$$

In case of spline model, the cubic polynomial associated to the first data subset ${\left\{y[n]\right\}}_{n=\bar{1,M_{1}}}$ is:

(4) $$y_{1}[n]=a_{1}+b_{1}n+c_{1}n^{2}+d_{1}n^{3},\;\forall n\in \bar{1,M_{1}}$$

with natural notations. The polynomial (4) can be expressed in linear regression form, with the regression vector $\varphi_{1}^{T}[n]=\left[\begin{array}{cccc} 1 & n & n^{2} & n^{3} \end{array}\right]$ and the unknown parameters vector $\theta_{1}^{4}=\left[\begin{array}{cccc} a_{1} & b_{1} & c_{1} & d_{1} \end{array}\right]$. In general, $\theta_{k}^{l}$ is the parameters vector corresponding to the k-th data subset and a polynomial of degree l−1.

In the first case, assume that the spline model has to be continuous and no conditions on derivatives are imposed. According to the general LS solution (3), the coefficients of the first polynomial in the spline curve are estimated as follows:

(5) $${\hat{\theta }}_{1}^{4}={\left[\sum_{n=1}^{M_{1}}n^{i+j-2}\right]}_{i,j=\bar{1,4}}^{-1}{\left[\sum_{n=1}^{M_{1}}n^{i-1}y[n]\right]}_{i=\bar{1,4}}$$

Thus, the first simulated polynomial naturally results in:

(6) $${\hat{y}}_{1}[n]=\varphi_{1}^{T}[n]{\hat{\theta }}_{1}^{4},\;\forall n\in \bar{1,M_{1}}.$$

This also defines the first knot, through which the next polynomial has to pass: $\left(M_{1},{\hat{y}}_{1}\left[M_{1}\right]\right)=\left(M_{1},\varphi_{1}^{T}\left[M_{1}\right]{\hat{\theta }}_{1}^{4}\right)$, in order to meet the continuity condition of the entire spline model.

Starting from the first polynomial, the next polynomial (associated to the second data subset) has to be determined by enforcing the continuity condition of spline model in the knot $\left(M_{1},{\hat{y}}_{1}\left[M_{1}\right]\right)$. This will reduce the number of coefficients to estimate by 1 with the help of LSM. Another reduction is obtained if a second knot is ad hoc defined, at the right side of the current subset, i.e., at the normalized instant $N_{2}^{e}=M_{1}+M_{2}$. More specifically, the knot can be defined by data averaging technique, as follows:

(7) $$\left(N_{2}^{e},\frac{y\left[N_{2}^{e}-1\right]+y\left[N_{2}^{e}\right]+y\left[N_{2}^{e}+1\right]}{3}\right)$$

If the polynomial $y_{2}$ has to pass through the knot (7) as well, then the number of parameters to estimate decreases from 4 to 2. This allows solving the LS problem in closed form, which speeds up the procedure of spline model construction. The averaging technique above is not the only one to define the new knot. Other techniques can be employed as well (even, for instance, by simply taking $y\left[N_{2}^{e}\right]$ instead of the average). Moreover, averaging can be performed with more than 3 successive data. In general, averaging has the advantage of not enforcing the spline curve to pass through measured data (which are corrupted by noises), while keeping its variation close to those data. The number of data to average allows the user to control the placement of knots with respect to raw data, depending on how strong the noise corrupting them is (the stronger the noise, the less data to average).

Consider the general instance of the (k−1)-th data subset (where k≥2) and assume the corresponding cubic polynomial is already identified. It is easy to see that the next data subset (the k-th one) begins at the normalized instant $N_{k}^{b}=M_{1}+M_{2}+\cdots +M_{k-1}+1=N_{k-1}^{e}+1$ and ends at the normalized instant $N_{k}^{e}=N_{k-1}^{e}+M_{k}$. The k-th cubic polynomial is then:

(8) $$y_{k}[n]=a_{k}+b_{k}n+c_{k}n^{2}+d_{k}n^{3},\;\forall n\in \bar{N_{k}^{b},N_{k}^{e}},$$

where $a_{k}$, $b_{k}$, $c_{k}$ and $d_{k}$ denote the unknown coefficients.

The model (8) has to be estimated subject to the following continuity constraints, imposed to the left and right side knots of current data subset:

(9) $$\{\begin{array}{l} y_{k}\left[N_{k}^{b}-1\right]=y_{\mathrm{left}}^{0}={\hat{y}}_{k-1}\left[N_{k}^{b}-1\right]={\hat{y}}_{k-1}\left[N_{k-1}^{e}\right]\\ y_{k}\left[N_{k}^{e}\right]=y_{\mathrm{right}}^{0}=\frac{y\left[N_{k}^{e}-1\right]+y\left[N_{k}^{e}\right]+y\left[N_{k}^{e}+1\right]}{3} \end{array}$$

(10) $$\{\begin{array}{l} a_{k}+b_{k}N_{k-1}^{e}+c_{k}{\left(N_{k-1}^{e}\right)}^{2}+d_{k}{\left(N_{k-1}^{e}\right)}^{3}=y_{\mathrm{left}}^{0}\\ a_{k}+b_{k}N_{k}^{e}+c_{k}{\left(N_{k}^{e}\right)}^{2}+d_{k}{\left(N_{k}^{e}\right)}^{3}=y_{\mathrm{right}}^{0} \end{array}$$

Next, by using the system (10), parameters $a_{k}$ and $b_{k}$ can be expressed as follows:

(11) $$\begin{array}{l} b_{k}=\frac{y_{\mathrm{right}}^{0}-y_{\mathrm{left}}^{0}}{M_{k}}-c_{k}\left(N_{k-1}^{e}+N_{k}^{e}\right)-d_{k}\frac{{\left(N_{k}^{e}\right)}^{3}-{\left(N_{k-1}^{e}\right)}^{3}}{M_{k}};\\ a_{k}=y_{\mathrm{left}}^{0}-b_{k}N_{k-1}^{e}-c_{k}{\left(N_{k-1}^{e}\right)}^{2}-d_{k}{\left(N_{k-1}^{e}\right)}^{3}=\frac{y_{\mathrm{left}}^{0}N_{k}^{e}-y_{\mathrm{right}}^{0}N_{k-1}^{e}}{M_{k}}+c_{k}N_{k-1}^{e}N_{k}^{e}+d_{k}N_{k-1}^{e}N_{k}^{e}\left(N_{k-1}^{e}+N_{k}^{e}\right). \end{array}$$

After inserting (11) in (8) and performing some algebraic manipulations, the optimization criterion (2) becomes:

(12) $$V_{k}\left(\theta_{k}^{2}\right)=\sum_{n=N_{k}^{b}}^{N_{k}^{e}}{\left(\tilde{y}[n]-\varphi_{k}^{T}[n]\theta_{k}^{2}\right)}^{2}$$

where:

(13) $$[\begin{array}{l} \tilde{y}[n]=y[n]-\frac{y_{\mathrm{left}}^{0}\left(N_{k}^{e}-n\right)-y_{\mathrm{right}}^{0}\left(N_{k-1}^{e}-n\right)}{M_{k}}\\ \varphi_{k}^{T}[n]=\left(N_{k}^{e}-n\right)\left(N_{k-1}^{e}-n\right)\left[\begin{array}{cc} 1 & N_{k-1}^{e}+N_{k}^{e}+n \end{array}\right]\\ \theta_{k}^{2}={\left[\begin{array}{cc} c_{k} & d_{k} \end{array}\right]}^{T} \end{array},\;\forall n\in \bar{N_{k}^{b},N_{k}^{e}}$$

With expression (3) of the LS solution, the unknown parameters $c_{k}$ and $d_{k}$ are identified as follows:

(14) $${\hat{\theta }}_{k}^{2}=\left[\begin{array}{c} {\hat{c}}_{k}\\ {\hat{d}}_{k} \end{array}\right]=R_{k}^{-1}r_{k}={\left[\begin{array}{cc} r_{k}^{11} & r_{k}^{12}\\ r_{k}^{21} & r_{k}^{22} \end{array}\right]}^{-1}\left[\begin{array}{c} r_{k}^{1}\\ r_{k}^{2} \end{array}\right]=\frac{1}{r_{k}^{11}r_{k}^{22}-r_{k}^{12}r_{k}^{21}}\left[\begin{array}{rr} r_{k}^{22} & -r_{k}^{12}\\ -r_{k}^{21} & r_{k}^{11} \end{array}\right]\left[\begin{array}{c} r_{k}^{1}\\ r_{k}^{2} \end{array}\right]=\frac{1}{r_{k}^{11}r_{k}^{22}-r_{k}^{12}r_{k}^{21}}\left[\begin{array}{c} r_{k}^{1}r_{k}^{22}-r_{k}^{2}r_{k}^{12}\\ r_{k}^{2}r_{k}^{11}-r_{k}^{1}r_{k}^{21} \end{array}\right]$$

where:

(15) $$[\begin{array}{l} r_{k}^{11}=\sum_{n=N_{k}^{b}}^{N_{k}^{e}}{\left(N_{k}^{e}-n\right)}^{2}{\left(N_{k-1}^{e}-n\right)}^{2}\\ r_{k}^{12}=r_{k}^{21}=\sum_{n=N_{k}^{b}}^{N_{k}^{e}}{\left(N_{k}^{e}-n\right)}^{2}{\left(N_{k-1}^{e}-n\right)}^{2}\left(N_{k-1}^{e}+N_{k}^{e}+n\right)\\ r_{k}^{22}=\sum_{n=N_{k}^{b}}^{N_{k}^{e}}{\left(N_{k}^{e}-n\right)}^{2}{\left(N_{k-1}^{e}-n\right)}^{2}{\left(N_{k-1}^{e}+N_{k}^{e}+n\right)}^{2}\\ r_{k}^{1}=\sum_{n=N_{k}^{b}}^{N_{k}^{e}}\left(N_{k}^{e}-n\right)\left(N_{k-1}^{e}-n\right)\tilde{y}[n]\\ r_{k}^{2}=\sum_{n=N_{k}^{b}}^{N_{k}^{e}}\left(N_{k}^{e}-n\right)\left(N_{k-1}^{e}-n\right)\left(N_{k-1}^{e}+N_{k}^{e}+n\right)\tilde{y}[n] \end{array}$$

Note that the coefficients $c_{k}$ and $d_{k}$ are estimated in closed form (it is not necessary to invert the matrix $R_{k}$ by numerical procedures). They allow estimating the coefficients $a_{k}$ and $b_{k}$ directly from (11).

The simulation expression of the cubic polynomial is then:

(16) $${\hat{y}}_{k}[n]=a_{k}+{\hat{b}}_{k}n+[n^{2}\;n^{3}]{\hat{\theta }}_{k}^{2},\;\forall n\in \bar{N_{k}^{b},N_{k}^{e}}$$

Consider now the second case, when continuity conditions are extended to the first derivative. Obviously, the first polynomial is identified by using Equations (4)–(6). The next polynomial has to continue the variation of previous polynomial and, moreover, to have the same first derivative value in the shared knot.

In the context above, one aims to determine the cubic polynomial (8) associated to the k-th data subset, subject to both continuity and derivability constraints enforced in the knot $\left(N_{k-1}^{e},{\hat{y}}_{k-1}\left[N_{k-1}^{e}\right]\right)$. According to the previous notations policy, hereafter, $y_{\mathrm{left}}^{1}$ will stand for the left side polynomial first derivative value at $N_{k-1}^{e}$ normalized instant. This value can be determined from the previously estimated model:

(17) $$y_{\mathrm{left}}^{1}={\hat{y}}_{k-1}^{\prime }[N_{k-1}^{e}]={\hat{b}}_{k-1}+2{\hat{c}}_{k-1}N_{k-1}^{e}+3d_{k-1}{\left(N_{k-1}^{e}\right)}^{2}$$

At this point, there is the possibility to replace the second condition in (9) by:

(18) $$y_{k}^{\prime }\left[N_{k-1}^{e}\right]=y_{\mathrm{left}}^{1}$$

which practically leads to the same computations as in the previous case. This approach is not recommended though, because of a bad and undesirable effect, especially induced when data are corrupted by strong noises: the spline curve might include high frequency oscillations. The main cause of this effect is the lack of conditions imposed at the right side of current subset.

Then, it is wiser to work with the following constraints:

(19) $$\{\begin{array}{l} y_{k}\left[N_{k}^{b}-1\right]=y_{\mathrm{left}}^{0}={\hat{y}}_{k-1}\left[N_{k}^{b}-1\right]={\hat{y}}_{k-1}\left[N_{k-1}^{e}\right]\\ \begin{array}{l} y_{k}\left[N_{k}^{e}\right]=y_{\mathrm{right}}^{0}=\frac{y\left[N_{k}^{e}-1\right]+y\left[N_{k}^{e}\right]+y\left[N_{k}^{e}+1\right]}{3}\\ {y^{\prime }}_{k}\left[N_{k-1}^{e}\right]=y_{\mathrm{left}}^{1}={{\hat{y}}^{\prime }}_{k-1}\left[N_{k-1}^{e}\right] \end{array} \end{array}$$

i.e., to join the conditions (9) and (18).

This time, an optimization problem with three constraints has to be solved. The constraints are expressed by the following linear system (as obtained from (8) and (19)):

(20) $$\{\begin{array}{l} a_{k}+b_{k}N_{k-1}^{e}+c_{k}{\left(N_{k-1}^{e}\right)}^{2}+d_{k}{\left(N_{k-1}^{e}\right)}^{3}=y_{\mathrm{left}}^{0}\\ \begin{array}{l} a_{k}+b_{k}N_{k}^{e}+c_{k}{\left(N_{k}^{e}\right)}^{2}+d_{k}{\left(N_{k}^{e}\right)}^{3}=y_{\mathrm{right}}^{0}\\ b_{k}+2c_{k}N_{k-1}^{e}+3d_{k}{\left(N_{k-1}^{e}\right)}^{2}=y_{\mathrm{left}}^{1} \end{array} \end{array}$$

From (20), the parameters $a_{k}$, $b_{k}$, $c_{k}$ can be expressed as functions of $d_{k}$. After few algebraic manipulations, their final expressions are obtained:

(21) $$\begin{array}{l} c_{k}=\frac{y_{\mathrm{right}}^{0}-y_{\mathrm{left}}^{0}}{M_{k}^{2}}-\frac{y_{\mathrm{left}}^{1}}{M_{k}}-d_{k}\left(2N_{k-1}^{e}+N_{k}^{e}\right);\\ b_{k}=y_{\mathrm{left}}^{1}\frac{N_{k-1}^{e}+N_{k}^{e}}{M_{k}}-2\frac{\left(y_{\mathrm{right}}^{0}-y_{\mathrm{left}}^{0}\right)N_{k-1}^{e}}{M_{k}^{2}}+d_{k}N_{k-1}^{e}\left(N_{k-1}^{e}+2N_{k}^{e}\right);\\ a_{k}=y_{\mathrm{left}}^{0}+\frac{\left(y_{\mathrm{right}}^{0}-y_{\mathrm{left}}^{0}\right){\left(N_{k-1}^{e}\right)}^{2}}{M_{k}^{2}}-y_{\mathrm{left}}^{1}\frac{N_{k-1}^{e}N_{k}^{e}}{M_{k}}-d_{k}{\left(N_{k-1}^{e}\right)}^{2}N_{k}^{e}. \end{array}$$

By inserting expressions (21) into definition (8) and suitably grouping the terms, it results that the polynomial model depends on $d_{k}$ parameter only:

(22) $$y_{k}[n]=y_{\mathrm{left}}^{0}+\frac{\left(y_{\mathrm{right}}^{0}-y_{\mathrm{left}}^{0}\right){\left(N_{k-1}^{e}-n\right)}^{2}}{M_{k}^{2}}-\frac{y_{\mathrm{left}}^{1}}{M_{k}}\left(N_{k-1}^{e}-n\right)\left(N_{k}^{e}-n\right)-d_{k}{\left(N_{k-1}^{e}-n\right)}^{2}\left(N_{k}^{e}-n\right),\;\forall \;n\in \bar{N_{k}^{b},N_{k}^{e}}.$$

With (22), the optimization criterion (2) becomes:

(23) $$V_{k}\left(\theta_{k}^{1}\right)=\sum_{n=N_{k}^{b}}^{N_{k}^{e}}{\left(\tilde{y}[n]-\varphi_{k}^{}[n]\theta_{k}^{1}\right)}^{2}$$

where:

(24) $$[\begin{array}{l} \tilde{y}[n]=y[n]-y_{\mathrm{left}}^{0}-\frac{\left(y_{\mathrm{right}}^{0}-y_{\mathrm{left}}^{0}\right){\left(N_{k-1}^{e}-n\right)}^{2}}{M_{k}^{2}}+\frac{y_{\mathrm{left}}^{1}}{M_{k}}\left(N_{k-1}^{e}-n\right)\left(N_{k}^{e}-n\right)\\ \varphi_{k}^{}[n]={\left(N_{k-1}^{e}-n\right)}^{2}\left(n-N_{k}^{e}\right)\;(scalar)\\ \theta_{k}^{1}=d_{k}\;(scalar) \end{array},\;\forall n\in \bar{N_{k}^{b},N_{k}^{e}}.$$

According to (3), the parameter $d_{k}$ is identified straightforwardly:

(25) $${\hat{d}}_{k}=\frac{\sum_{n=N_{k}^{e}}^{N_{k}^{b}}{\left(N_{k-1}^{e}-n\right)}^{2}\left(n-N_{k}^{e}\right)\tilde{y}[n]}{\sum_{n=N_{k}^{e}}^{N_{k}^{b}}{\left(N_{k-1}^{e}-n\right)}^{4}{\left(n-N_{k}^{e}\right)}^{2}}$$

The other three parameters are then estimated by inserting (25) into (21). The simulation model is finally obtained by using the estimates of four parameters in (8).

To conclude the subsection, the mechanism of subset enhancement is explained next. As already stated, each time a new output raw value joins the current subset, the length $M_{k}$ is incremented by 1. It has to be outlined that the ad hoc created knots (such as (7)) do not necessarily belong to data subsets. Such knots are only employed to express the concatenation conditions of successive cubic polynomials that constitute the spline model.

Incrementing $M_{k}$ automatically involves determining a new estimation of corresponding polynomial, since the end normalized instant $N_{k}^{e}$ is incremented as well. Assume the cubic polynomial has been identified (as previously described). Then, the question is: how to stop the subset enhancement? The standard deviation (std) of error between the subset data and the simulated identified polynomial can serve as a stop test. In the current framework, this statistical parameter is defined as follows:

(26) $$\sigma_{y_{k}}=\sqrt{\frac{1}{M_{k}}\sum_{n=N_{k}^{b}}^{N_{k}^{e}}{\left(y[n]-{\hat{y}}_{k}[n]\right)}^{2}}=\sqrt{\frac{1}{M_{k}}V_{k}\left({\hat{\theta }}_{k}^{4}\right)}$$

One can see that solving the identification problem actually means not only estimating the optimal cubic polynomial, but also evaluating its performance given by the minimum value of quadratic criterion. In this way, the std is easy to compute, with minimum effort. 

For each data subset, after estimating the two cubic polynomials (without and with derivative constraints), they are compared in terms of std (26). The best of them is the one with the smallest std. Naturally, during the subset enhancement, the std exhibits variations. If, for both optimal polynomials, the std has successively increased a number of times, M (e.g., M=5), the enhancement is stopped. After that, $M_{k}$ is decremented by M. The best corresponding polynomial is then selected according to the final data subset, of updated length $M_{k}$.

The procedure of building the stochastic cubic spline model is summarized in Appendix A.

### 2.2. Identifying the Multivariable Models

Consider a *Multi-Input Multi-Output* (MIMO) process with nu×ny I/O channels. Assume that this multivariable process can provide ny I/O measured data sets of length $N\in {\mathbb{N}}^{*},$ namely $D_{N,j}={\left\{u_{1}[n],u_{2}[n],\ldots ,u_{nu}[n],y_{j}[n]\right\}}_{n\in \bar{1,N}}$, where $j\in \bar{1,ny}$. In general, identification of MIMO systems is a difficult task, even the number of I/O channels is not so large. Therefore, instead of identifying the whole MIMO system, the data subsets above can be employed separately, in order to identify one *Multi-Input Single-Output* (MISO) subprocess at a time, corresponding to each output. Consider then any of the MISO subprocesses included into the MIMO process. To keep the subsequent explanation as simple as possible, the output signal is hereafter denoted by y (instead of $y_{j}$).

The useful filter of identification model relies on the MISO ARX model, as follows:

(27) $$A\left(q^{-1}\right)y[n]=B_{1}\left(q^{-1}\right)u_{1}[n]+\cdots +B_{nu}\left(q^{-1}\right)u_{nu}[n]+v[n],\;\forall n\in \bar{1,N}.$$

In (27), $q^{-1}$ stands for the one step delay operator ($\left(q^{-1}f\right)[n]=f[n-1]$, ∀n∈ℤ), v is the colored noise that directly affects the output data y, and A, $B_{1}$, …, $B_{nu}$ are polynomials expressed as:

(28) $$\left\{ \begin{array}{l} A\left(q^{-1}\right)=1+a_{1}q^{-1}+\cdots +a_{na}q^{-na}\\ B_{1}\left(q^{-1}\right)=q^{1-nk_{1}}\left(b_{1,1}q^{-1}+b_{2,1}q^{-2}\cdots +b_{nb_{1},1}q^{-nb_{1}}\right)\\ B_{2}\left(q^{-1}\right)=q^{1-nk_{2}}\left(b_{1,2}q^{-1}+b_{2,2}q^{-2}\cdots +b_{nb_{2},2}q^{-nb_{2}}\right)\\ \;\vdots \\ B_{nu}\left(q^{-1}\right)=q^{1-nk_{nu}}\left(b_{1,nu}q^{-1}+b_{2,nu}q^{-2}\cdots +b_{nb_{nu},nu}q^{-nb_{nu}}\right) \end{array}\right.$$

Assuming that the polynomial degrees na, $nb_{1}+nk_{1}$, …, $nb_{nu}+nk_{nu}$, and the intrinsic delays ${\left\{nk_{i}\right\}}_{i=\bar{1,nu}}$ (usually, unitary), i.e., the model structural indices in (28), are known, the coefficients of the ARX model can be estimated by means of LSM [12,13].

The simulated output of the useful component $y_{\mathrm{ARX}}$ can be computed as follows:

(29) $$\begin{array}{ll} y_{\mathrm{ARX}}[n]= & -{\hat{a}}_{1}y_{\mathrm{ARX}}[n-1]-{\hat{a}}_{2}y_{\mathrm{ARX}}[n-2]-\cdots -{\hat{a}}_{na}y_{\mathrm{ARX}}[n-na]+\\ & +{\hat{b}}_{1,1}u_{1}\left[n-nk_{1}\right]+\cdots +{\hat{b}}_{nb_{1},1}u_{1}\left[n-nk_{1}-nb_{1}+1\right]+\\ & +{\hat{b}}_{1,2}u_{2}\left[n-nk_{2}\right]+\cdots +{\hat{b}}_{nb_{2},2}u_{2}\left[n-nk_{2}-nb_{2}+1\right]+\cdots +\\ & +{\hat{b}}_{1,nu}u_{nu}\left[n-nk_{nu}\right]+\cdots +{\hat{b}}_{nb_{nu},nu}u_{nu}\left[n-nk_{nu}-nb_{nu}+1\right],\;\;\forall n\in \bar{1,N}, \end{array}$$

where, the symbol $\hat{\cdot }$ stands for the estimated parameters of ARX model (after using LSM). A natural initialization of recursive Equation (29) is $y_{\mathrm{ARX}}[n]=y[n]$, for each $n\in \bar{1,\max\left\{na,nb_{1}\ldots nb_{nu}\right\}}$.

After subtracting the simulated output $y_{ARX}$ (i.e., the useful data) from the measured data y, a residual noise $v_{r}$ is obtained:

(30) $$v_{r}[n]=y[n]-y_{\mathrm{ARX}}[n],\;\forall n\in \bar{1,N}$$

Then, the noise filter can be identified by using noisy data (30) and the ARMA model:

(31) $$\left\{ \begin{array}{l} v_{r}[n]=\frac{C\left(q^{-1}\right)}{D\left(q^{-1}\right)}e[n]\\ E\left\{e[n]e[m]\right\}=\lambda^{2}\delta_{0}[n-m] \end{array}\right.,\;\forall n\in \bar{1,N}$$

where e is a Gaussian white noise with unknown variance $\lambda^{2}$, whilst C and D are polynomials of degrees nc and nd, respectively:

(32) $$\left\{ \begin{array}{l} C\left(q^{-1}\right)=1+c_{1}q^{-1}+\cdots +c_{nc}q^{-nc}\\ D\left(q^{-1}\right)=1+d_{1}q^{-1}+\cdots +d_{nd}q^{-nd} \end{array}\right.$$

If the structural indices of the model in (31) and (32) are known, the coefficients of the ARMA model can be estimated by using the *Minimum Prediction Error Method* (MPEM) [12,13], based on the quadratic criterion. After estimating the polynomials in (32), the simulated noise $v_{\mathrm{ARMA}}$ results from the system below:

(33) $$\left\{ \begin{array}{l} v_{\mathrm{ARMA}}[n]=-{\hat{d}}_{1}v_{\mathrm{ARMA}}[n-1]-\cdots -{\hat{d}}_{nd}v_{\mathrm{ARMA}}[n-nd]+{\hat{c}}_{1}\hat{e}[n-1]+\cdots +{\hat{c}}_{nc}\hat{e}[n-nc]\\ \hat{e}[n]=v_{r}[n]+{\hat{\alpha }}_{1}v_{r}[n-1]+\cdots +{\hat{\alpha }}_{n\alpha }v_{r}[n-n\alpha ] \end{array}\right.,\;\forall n\in \bar{1,N}$$

where $\hat{e}$ is the estimation of the white noise obtained by using an approximant AR model of order nα, which has to be set much bigger than max{nc,nd}. The recursive procedure (33) starts from the natural initialization: $v_{\mathrm{ARMA}}[n]=v_{r}[n]$, $\forall n\in \bar{1,\max\{nc,nd\}}$.

Finally, the estimation of the residual white noise is obtained by:

(34) $$\varepsilon [n]=v_{r}[n]-v_{\mathrm{ARMA}}[n],\;\forall \;n\in \bar{1,N}$$

The full identification of ARX-ARMA models (27) and (31) requires solving a granular optimization problem [25], as the structural indices are unknown. In order to find the optimal structure of ARX-ARMA models, two approaches are considered, as explained next. 

Firstly, a two-step optimization problem can be solved, as follows: (a) find the optimal structural indices $\left\{na,nb_{1},\ldots ,nb_{nu}\right\}$ for ARX model (27); (b) find the optimal structural indices {nc,nd,nα} for the couple of ARMA-AR models (see the system (33)), by taking into account the simulated output of the previously obtained optimal ARX model $y_{\mathrm{ARX}}$ (see (29)) and more specifically, the resulting noise $v_{r}$ (estimated as in (30)). Obviously, this method yields two independent optimal models: one providing the useful information extracted from data and one dealing with the noise corrupting the data. 

Secondly, a global optimization problem can be solved. This time, the optimal structural indices of all models, namely $\left\{na,nb_{1},\ldots ,nb_{nu},nc,nd,n\alpha \right\}$, have to be found. Thus, a global (overall) optimal model will result. In this aim, the optimization criterion relies on the overall simulated output below:

(35) $$y_{\mathrm{ovr}}[n]=y_{\mathrm{ARX}}[n]+v_{\mathrm{ARMA}}[n],\;\forall n\in \bar{1,N}$$

Accordingly, the residual white noise is:

(36) $$\varepsilon_{\mathrm{ovr}}[n]=y[n]-y_{\mathrm{ovr}}[n],\;\forall n\in \bar{1,N}$$

In the SI community, it is well known that any identification model has to be validated. This means stimulating both the model and the associated process by different inputs from the ones employed to obtain the identification data set and comparing the resulted outputs. Usually, such a comparison is performed according to some *whitening* tests [12,13] (i.e., the difference between simulated and acquired outputs should have the characteristics of a white noise).

In the current framework, two data sets can be acquired from the start: one for *identification*, $D_{N}^{\mathrm{id}}$, and another one for *validation*, $D_{N}^{\mathrm{va}}$. More specifically:

(37) $$[\begin{array}{l} D_{N}^{\mathrm{id}}={\left\{u_{1}^{\mathrm{id}}[n],u_{2}^{\mathrm{id}}[n],\ldots ,u_{nu}^{\mathrm{id}}[n],y^{\mathrm{id}}[n]\right\}}_{n\in \bar{1,N}}\\ D_{N}^{\mathrm{va}}={\left\{u_{1}^{\mathrm{va}}[n],u_{2}^{\mathrm{va}}[n],\ldots ,u_{nu}^{\mathrm{va}}[n],y^{\mathrm{va}}[n]\right\}}_{n\in \bar{1,N}} \end{array}$$

The optimization criterion employed to determine the structural indices has to account both acquired data sets (37), with some weights, depending on which one of them is more important. Usually, identification and validation are equally important. In this manner, the optimal models are simultaneously identified and validated.

Come back now to the granular optimization problem. In the first approach, determination of ARX model (27) and (28) with known structural indices follows the strategy below:

1. Identify the ARX model from the identification data set $D_{N}^{\mathrm{id}}$, by means of LSM.
2. Generate the useful signals $y_{\mathrm{ARX}}^{\left\{\mathrm{id},\mathrm{va}\right\}}$ by using (29) and $D_{N}^{\left\{\mathrm{id},\mathrm{va}\right\}}$, respectively.
3. Compute the residual colored noises $v_{r}^{\left\{\mathrm{id},\mathrm{va}\right\}}$ by using (30), $y_{\mathrm{ARX}}^{\left\{\mathrm{id},\mathrm{va}\right\}}$ and $D_{N}^{\left\{\mathrm{id},\mathrm{va}\right\}}$, respectively.

In order to find the optimal structure of ARX model, the following criterion can be maximized (based on SNR):

(38) $${\mathrm{SNR}}_{\mathrm{ARX}}\left[na,nb_{1},\ldots ,nb_{nu}\right]=\lambda \;{\mathrm{SNR}}_{\mathrm{ARX}}^{\mathrm{id}}\left[na,nb_{1},\ldots ,nb_{nu}\right]+(1-\lambda )\;{\mathrm{SNR}}_{\mathrm{ARX}}^{\mathrm{va}}\left[na,nb_{1},\ldots ,nb_{nu}\right]$$

where λ∈[0,1] (weight), and:

(39) $${\mathrm{SNR}}_{\mathrm{ARX}}^{\left\{\mathrm{id},\mathrm{va}\right\}}\left[na,nb_{1},\ldots ,nb_{nu}\right]=20\log\left(\frac{\sigma_{y^{\left\{\mathrm{id},\mathrm{va}\right\}}}}{\sigma_{v_{r}^{\left\{\mathrm{id},\mathrm{va}\right\}}}}\right)$$

(expressed in dB). In definitions (39), $\sigma_{y^{\left\{\mathrm{id},\mathrm{va}\right\}}}$ and $\sigma_{v_{r}^{\left\{\mathrm{id},\mathrm{va}\right\}}}$ stand for the std of corresponding signals. Although not explicitly written in (39), both stds depend on structural indices of ARX model. Recall that, if x is a N-length digital signal, then its std is defined as follows:

(40) $$\sigma_{x}=\sqrt{\frac{1}{N}\sum_{n=1}^{N}{\left(x[n]-\mu_{x}\right)}^{2}},\;\mathrm{with}\;\mu_{x}=\frac{1}{N}\sum_{n=1}^{N}x[n]$$

The ARMA model (31) can similarly be determined (by continuing the strategy above):

4. Identify the ARMA model from the residual noise $v_{r}^{\mathrm{id}}$, by means of MPEM.
5. Identify the AR model of white noise (see the second equation in (33)) from the residual noise $v_{r}^{\mathrm{id}}$, by means of LSM or Levinson-Durbin Algorithm [12,13].
6. Generate the noisy signals $v_{\mathrm{ARMA}}^{\left\{\mathrm{id},\mathrm{va}\right\}}$ by using (33) and $v_{r}^{\left\{\mathrm{id},\mathrm{va}\right\}}$, respectively.
7. Compute the residual white noises $\varepsilon^{\left\{\mathrm{id},\mathrm{va}\right\}}$ by using (34), $v_{r}^{\left\{\mathrm{id},\mathrm{va}\right\}}$ and $v_{\mathrm{ARMA}}^{\left\{\mathrm{id},\mathrm{va}\right\}}$, respectively.

The optimal structure of ARMA model can be found by maximizing the following optimization criterion:

(41) $${\mathrm{SNR}}_{\mathrm{ARMA}}\left[nc,nd,n\alpha \right]=\lambda \;{\mathrm{SNR}}_{\mathrm{ARMA}}^{\mathrm{id}}\left[nc,nd,n\alpha \right]+(1-\lambda )\;{\mathrm{SNR}}_{\mathrm{ARMA}}^{\mathrm{va}}\left[nc,nd,n\alpha \right]$$

where λ∈[0,1] and:

(42) $${\mathrm{SNR}}_{\mathrm{ARMA}}^{\left\{\mathrm{id},\mathrm{va}\right\}}\left[nc,nd,n\alpha \right]=20\log\left(\frac{\sigma_{v_{r}^{\left\{\mathrm{id},\mathrm{va}\right\}}}}{\sigma_{\varepsilon^{\left\{\mathrm{id},\mathrm{va}\right\}}}}\right)$$

In the second approach, identification of ARX and ARMA-AR models is performed at once. More specifically, the two procedures above are concatenated, resulting in a 7-step procedure. Nevertheless, the last step has to be replaced by:

8. Compute the residual white noises $\varepsilon_{\mathrm{ovr}}^{\left\{\mathrm{id},\mathrm{va}\right\}}$ by using (35), (36), $y_{\mathrm{ARX}}^{\left\{\mathrm{id},\mathrm{va}\right\}}$ and $v_{\mathrm{ARMA}}^{\left\{\mathrm{id},\mathrm{va}\right\}}$, respectively.

The optimal global model can be found by maximizing the following criterion:

(43) $$\begin{array}{ll} {\mathrm{SNR}}_{\mathrm{ovr}}\left[na,nb_{1},\ldots ,nb_{nu},nc,nd,n\alpha \right]= & \lambda {\mathrm{SNR}}_{\mathrm{ovr}}^{\mathrm{id}}\left[na,nb_{1},\ldots ,nb_{nu},nc,nd,n\alpha \right]+\\ & +(1-\lambda ){\mathrm{SNR}}_{\mathrm{ovr}}^{\mathrm{va}}\left[na,nb_{1},\ldots ,nb_{nu},nc,nd,n\alpha \right], \end{array}$$

where λ∈[0,1], and:

(44) $${\mathrm{SNR}}_{\mathrm{ovr}}^{\left\{\mathrm{id},\mathrm{va}\right\}}\left[na,nb_{1},\ldots ,nb_{nu},nc,nd,n\alpha \right]=20\log\left(\frac{\sigma_{y^{\left\{\mathrm{id},\mathrm{va}\right\}}}}{\sigma_{\varepsilon_{\mathrm{ovr}}^{\left\{\mathrm{id},\mathrm{va}\right\}}}}\right)$$

Usually, when noisy I/O data are employed in identification, the optimization criteria (38), (41) and (43) exhibit irregular variations, with many local extremes and derivative ruptures, due to the stochastic nature of noises corrupting the data. Consequently, in order to solve the SNR maximization problem, exact optimization methods [27] are useless. Hopefully, this type of problems can be solved by means of evolutionary computing techniques, referred to as *metaheuristics* [24,25]. Such a metaheuristic optimization technique is based on the classical *Hill Climbing Algorithm* (HCA). Within the next subsection, a modified and advanced version of this algorithm is described at length.

### 2.3. Advanced Hill Climbing

#### 2.3.1. Hill Climbing Principles

In the original version, the HCA was introduced as a local metaheuristic [24,25]. This means the search for the optimum is only performed in a reduced subset of search space, where the global optimum is very likely to lie. Nevertheless, the HCA can be modified to perform the search over the entire space. 

Two modified versions of classical HCA were introduced in [25]. Basically, the HCA was improved in two respects. Firstly, instead of working with a single climber (alpinist), a group of climbers is now activated to search for the optimum all over the space. Secondly, instead of advancing towards the optimum following the Monte-Carlo technique (i.e., blindly, without any strategy), each (virtual) climber receives a *compass*, helping it to choose the next position to approach. The compass actually is an estimation of movement gradient, computed by accounting the path the climber follows. In [28], the HCA employs a single climber endowed with a compass, in order to solve a multi-model identification problem. Hereafter, an *Advanced HCA* (AHCA) is introduced.

The specific mechanism of AHCA can briefly be described as follows: a whole group of climbers tries to reach for the highest peak of an irregular mountain (with many local peaks); each one starts climbing from a randomly chosen position, without being aware of the path to follow; however, each climber uses a compass to advance and, moreover, has the possibility to fly towards a very different position, when stuck on a dead end path. At any time, one of the climbers finds itself into the highest position. If, after a number of climbing iterations (e.g., 20), the highest position remains unchanged or if the total number of climbing iterations overcomes a threshold (e.g., 30 per climber), then the search is stopped and the highest climber in the group, together with its altitude, constitute the optimal solution found. The optimization criterion is then the altitude of a climber, which has to be maximized. The altitude can be measured by means of a cost function that plays the role of altimeter. For example, any criterion of the previous subsection, relying on SNR, can be an altimeter.

The main difference between the AHCA and the other HCAs (e.g., from [25] or [28]) is given by the possibility to control, to some extent, the trade-off between *exploration* and *exploitation*. According to evolutionary computing terminology, an efficient metaheuristic should be able both to *explore* as much as possible of the search space and to focus on (or to *exploit*) subsets of the search space (where the optimum seems to exist). In addition, it is suitable that the user can control how much time is allocated to each one of the two operations. Spending too much time in exploration might lead to slow convergence towards the optimum (even to oscillations between several possible optimal points). Focusing too much on a narrow subset might lead (even very fast) to a local optimum, missing the global one. Therefore, a balance between exploration and exploitation is necessary.

In case of AHCA, the trade-off exploration-exploitation can partially be controlled by introducing an operation to be applied when climbers are firmly stuck on local peaks, namely *the mutation*. The operation was inspired by Genetic Algorithms [25,29], and is explained next. 

If n is an integer and $b_{n}$ is the binary representation of n, then a *mutated* version of n is obtained as follows: (a) randomly select some of the bits in $b_{n}$; (b) mutate (change) the values of selected bits (0 becomes 1 and 1 becomes 0); (c) compute the new integer from the mutated binary representation. 

In the framework of AHCA, mutation is applied to those (virtual) climbers that are unable to move from their position to a higher one, after several trials. This allows them to fly towards a different position (from which they could continue climbing) and, thus, to avoid being stuck on local peaks. Consequently, exploration of search space is kept alive, given that, in general, AHCA rather is an exploitation procedure. 

#### 2.3.2. Advanced Hill Climbing Algorithm

**Figure 1:**
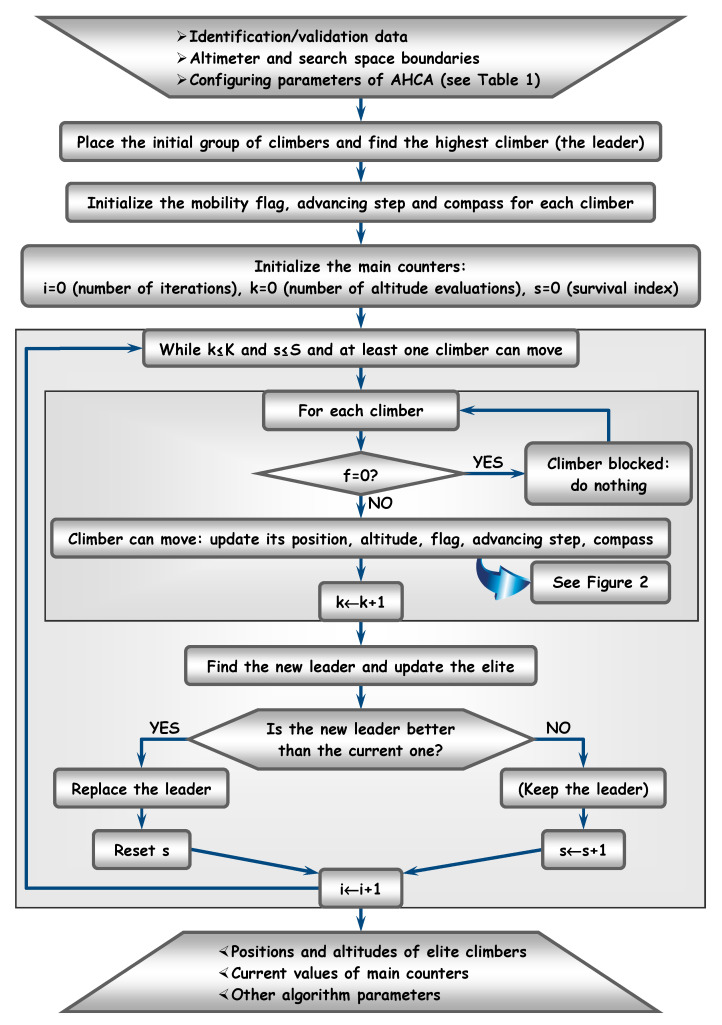
Flow diagram of the Advanced Hill Climbing Algorithm (AHCA).

**Figure 2:**
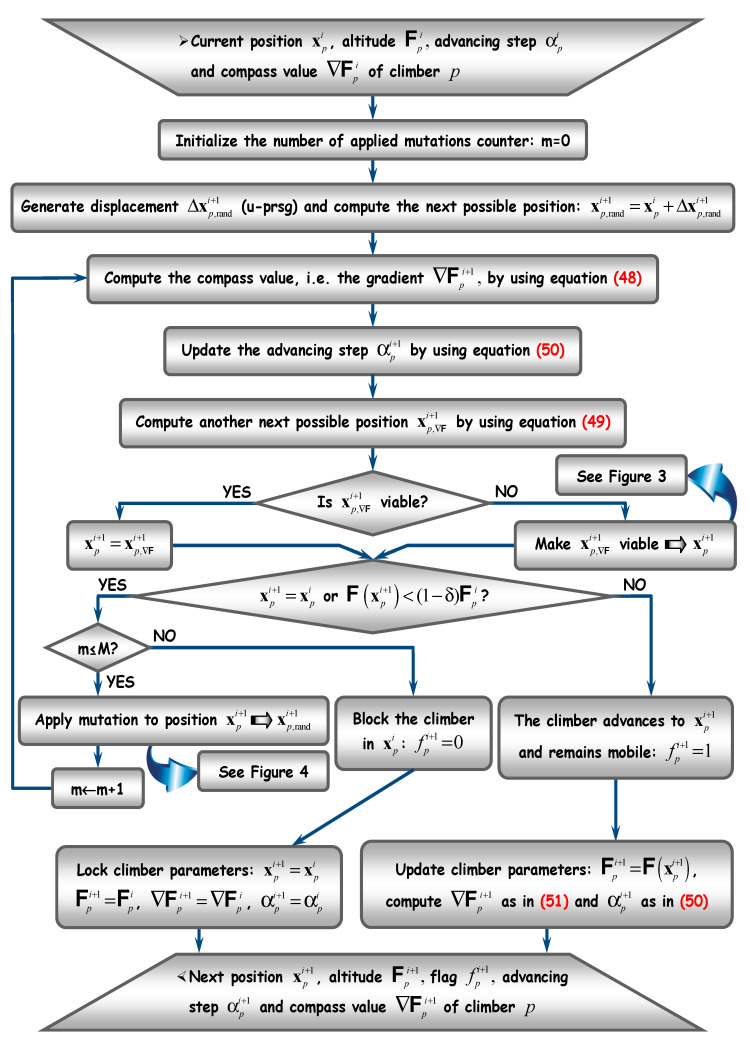
Upgrading procedure for climber parameters: position, altitude, flag, advancing step and compass.

**Figure 3:**
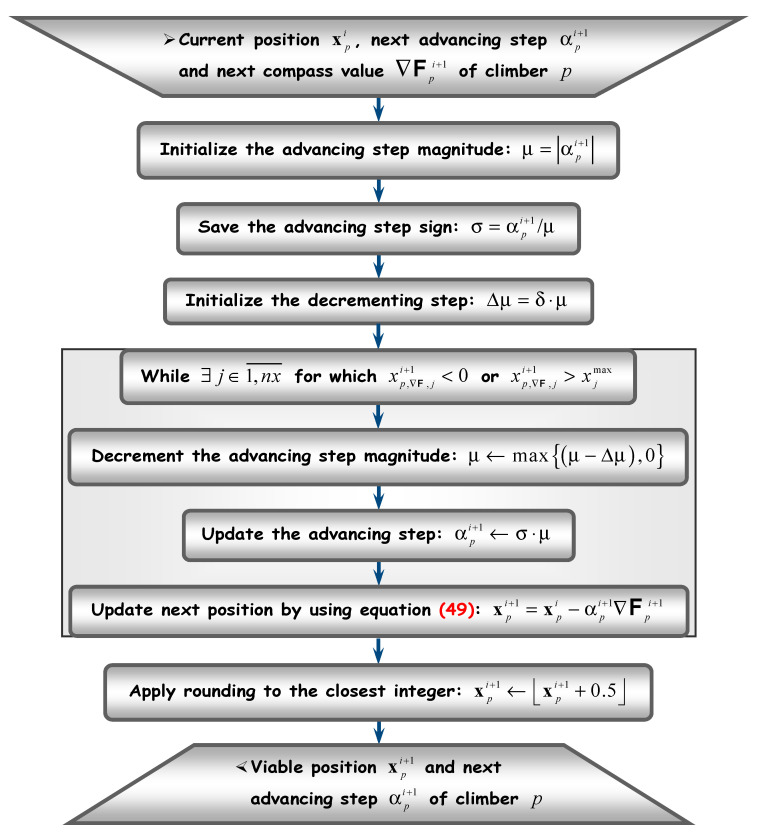
Making a climber position viable.

**Figure 4:**
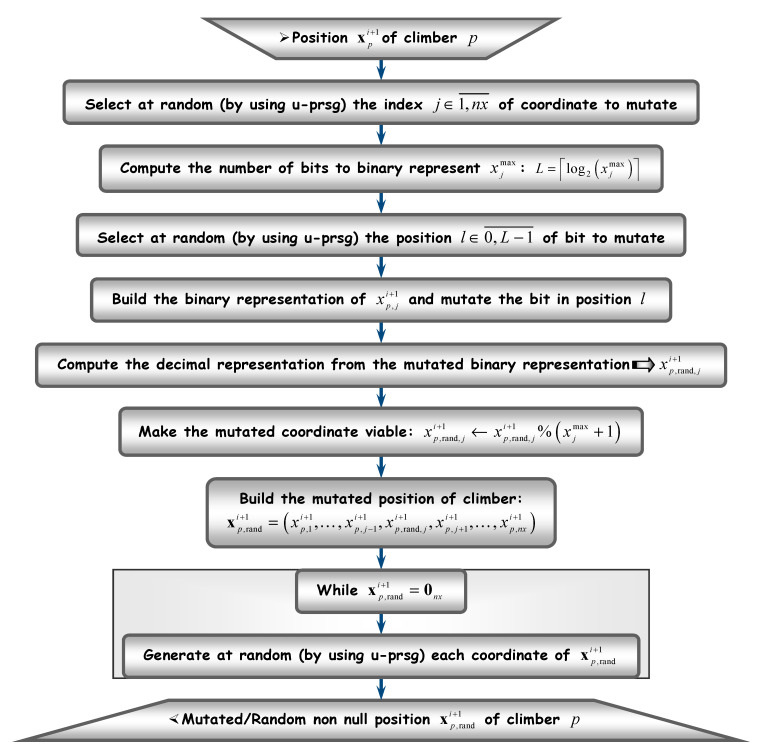
Mutating a climber position.

Figure 1, Figure 2, Figure 3 and Figure 4 depict the flow diagrams of AHCA, which are explained next at length (following each figure).

The algorithm requires a set of input parameters, in order to run (see the top of Figure 1). The numerical data can be I/O acquired signals, as previously explained. Let 𝗙 be the altimeter to maximize and denote by $𝗦\subseteq {\mathbb{R}}^{nx}$ the finite search space, of size $nx\in {\mathbb{N}}^{*}$. Assume the journey starts with a group (population) of $P\in {\mathbb{N}}^{*}$ climbers. At each iteration index, i∈ℕ, the climbers are placed in positions ${\left\{x_{p}^{i}\right\}}_{p\in \bar{1,P}}\subset 𝗦$. The altimeter can be one of the criteria (38), (41), (43). Accordingly, the generic position of a climber can be defined by the corresponding structural indices:

(45) $$[\begin{array}{l} x=\left(na,nb_{1},\ldots ,nb_{nu}\right)\in {\mathbb{N}}^{nu+1}\\ x=\left(nc,nd,n\alpha \right)\in {\mathbb{N}}^{3}\\ x=\left(na,nb_{1},\ldots ,nb_{nu},nc,nd,n\alpha \right)\in {\mathbb{N}}^{nu+4} \end{array}$$

Note that all positions (45) have integer coordinates. This feature enforces climbers to advance in positions with integer coordinates only. The boundaries of search space 𝗦 are defined by setting reasonable variation ranges of structural indices. For example, upper limits can be set from few tens to one hundred.

The configuring parameters are listed in Table 1, together with their default values. Beside the climbers group size, P, thresholds such as K, S or M can help the user to stop the search (as no convergence results were proven for metaheuristics like HCA) or to block climbers.

In addition, it is necessary to clearly discriminate between altitudes. Thus, if the relative difference between two altitudes is at most equal to δ, then the altitudes are considered equal. More specifically, if:

(46) $$\left|{𝗙}_{1}-{𝗙}_{2}\right|\leq \delta \left|{𝗙}_{1}\right|$$

Then one considers that ${𝗙}_{1}≅{𝗙}_{2}$. Finally, $P_{e}$ is the number of the best climbers (including the leader), which can constitute a special group, referred to as *elite*. In general, it is recommended to analyze not only the best solution returned by a metaheuristic, but also some good candidates as well. They could help the user to select better optimal points, with similar values of cost function, according to additional criteria. For example, in the case of identification models, if some of the elite climbers are located close to the leader (in altitude), then one can choose the climber that leads to the minimum number of poles, instead of the leader itself.

By convention, all input parameters of AHCA main procedure are *global*. This means they can be used inside any invoked subroutine, without passing them as input parameters of that subroutine.

Initially, all climbers are placed at random into starting positions, ${\left\{x_{p}^{0}\right\}}_{p\in \bar{1,P}}\subset 𝗦$. In this aim, as well as for further selections performed *at random*, a *uniformly distributed pseudo-random sequences generator* (u-prsg) can be used. To each climber, $p\in \bar{1,P}$, a mobility flag is assigned. Its value, $f_{p}^{i}$, is updated at each iteration. The climber can only continue moving on its path if $f_{p}^{i}$ is non null, otherwise it is blocked (probably on a local or even on the global peak). Initially, all climbers are free to move ($f_{p}^{0}=1$, $\forall p\in \bar{1,P}$).

The algorithm core is constituted by the climber’s position upgrading. This is a procedure that takes into account the compass information and requires initializing not only the compass values, but also the advancing steps for each climber, as it will be explained later.

Figure 1 reveals that the new position, altitude, flag, advancing step and compass are updated only for climbers that can move. Blocked climbers are not processed anymore. Each time the climbers group advances towards new (higher) positions, the elite is updated and even the leader can be changed. If the leader succeeds to keep its position (i.e., *survived*), the survival index is incremented. Otherwise, the index is reset for the new leader. After fulfilling the procedure termination condition, the elite performance (including the optimal solution), the final values of main counters and some algorithm characteristics are returned (e.g., the number of iterations, the running duration, the total number of arithmetic operations, the number of blocked climbers, etc.).

Focus now on Figure 2, which explains the upgrading manner of climber positions. For each climber, the next position is computed through a numerical procedure inspired by Cauchy’s gradient technique, with variable advancing step [27]. Assume the run is at iteration i∈ℕ and the next possible position of climber $p\in \bar{1,P}$ is:

(47) $$x_{p,\mathrm{rand}}^{i+1}=x_{p}^{i}+\Delta x_{p,\mathrm{rand}}^{i+1}$$

where the displacement $\Delta x_{p,\mathrm{rand}}^{i+1}$ is generated through the u-prsg. Then, a possible direction to follow is pointed by the climber compass, which actually depends on the gradient:

(48) $$\nabla {𝗙}_{p}^{i+1}={\left[\frac{𝗙\left(x_{p,\mathrm{rand}}^{i+1}\right)-𝗙\left(x_{p}^{i}\right)}{x_{p,\mathrm{rand},1}^{i+1}-x_{p,1}^{i}}\;\cdots \;\frac{𝗙\left(x_{p,\mathrm{rand}}^{i+1}\right)-𝗙\left(x_{p}^{i}\right)}{x_{p,\mathrm{rand},nx}^{i+1}-x_{p,nx}^{i}}\right]}^{T}$$

where, by convention, any null denominator is setting to null the corresponding component. According to Cauchy’s procedure, a second possible future position of climber is computed by using the compass:

(49) $$x_{p,\nabla 𝗙}^{i+1}=x_{p}^{i}-\alpha_{p}^{i+1}\nabla {𝗙}_{p}^{i+1}$$

where the advancing step has to be updated previously:

(50) $$\alpha_{p}^{i+1}=\alpha_{p}^{i}+{\left[\nabla {𝗙}_{p}^{i+1}\right]}^{T}\nabla {𝗙}_{p}^{i}$$

with $\alpha_{p}^{0}=1$ and $\nabla {𝗙}_{p}^{0}=0_{nx}$. Obviously, in (50), the gradient $\nabla {𝗙}_{p}^{i}$ is computed by taking into account the former position $x_{p}^{i-1}$ of the climber:

(51) $$\nabla {𝗙}_{p}^{i}={\left[\frac{𝗙\left(x_{p}^{i}\right)-𝗙\left(x_{p}^{i-1}\right)}{x_{p,1}^{i}-x_{p,1}^{i-1}}\;\cdots \;\frac{𝗙\left(x_{p}^{i}\right)-𝗙\left(x_{p}^{i-1}\right)}{x_{p,nx}^{i}-x_{p,nx}^{i-1}}\right]}^{T}$$

Before anything else, the position $x_{p,\nabla 𝗙}^{i+1}$ has to be made viable. This means first to bring back the result into the search space 𝗦, if jumped away. Then, the viable position needs rounding to the closest integer. Making the operation viable corresponds to the flow diagram in Figure 3 and will be described later.

Let $x_{p}^{i+1}$ be the viable next position. Then, it is suitable that $x_{p}^{i+1}\neq x_{p}^{i}$ and $x_{p}^{i+1}$ lies at an altitude at least equal to the current one, i.e.,: $𝗙\left(x_{p}^{i+1}\right)\geq (1-\delta )𝗙\left(x_{p}^{i}\right)$ (see (46)), if possible. Failing to meet this requirement involves applying mutation to $x_{p}^{i+1}$, in the hope that the climber will fly to a better position. Mutation can be applied according to the procedure in Figure 4, which will be described later. If, after several mutations, the position does not improve, then the climber has to be blocked on the current position $x_{p}^{i}$ and its parameters are locked. On the contrary, if $x_{p}^{i+1}$ becomes a better position than $x_{p}^{i}$, then the climber advances to it (while remaining mobile) and all its parameters have to be updated, as shown in the lower part of Figure 2.

Come back now to the operation of making a position viable. This is performed through the procedure illustrated in Figure 3, according to the following general principle: since the new position $x_{p,\nabla 𝗙}^{i+1}$ is obtained from the current position $x_{p}^{i}$ by moving along a direction pointed by the compass (i.e., by the gradient, $\nabla {𝗙}_{p}^{i+1}$ see (49)), one factor that pushed it away from the search space is the advancing step $\alpha_{p}^{i+1}$; it suffices then to gradually lower the magnitude of this step (while keeping the same direction), until the new position enters the search space.

Assume the search space boundaries are denoted by ${\left\{x_{j}^{\max}\right\}}_{j\in \bar{1,nx}}\subset {\mathbb{N}}^{*}$. As already mentioned, they are known at the entry level of procedure in Figure 3. The gradient direction is kept unchanged by only decreasing the magnitude of advancing step $\alpha_{p}^{i+1}$. This is why its sign has to be saved. The decrementing step is defined here by means of the accuracy threshold δ (a global parameter as well), although other choices would be possible. Note that, in order to preserve the advancing direction (as pointed by the compass), the rounding operation is solely applied in the end.

To conclude this subsection, let us explain next how the mutation can be applied. The corresponding numerical procedure is summarized in Figure 4. The main idea is that mutation should not make the climber fly too far away from the possible next viable position. Otherwise, the effort to reach the current altitude can be cancelled, which might involve a loss in the search speed. Therefore, mutation is applied to one of the position coordinates, selected at random. Furthermore, one single bit (also chosen at random) is mutated. In Figure 4, the bit to mutate lies in position $l\in \bar{0,L-1}$, where the bits are located from 0 (the least significant) to L−1 (the most significant). After applying mutation, the position is made viable by computing the remainder between the mutated coordinate ($x_{p,\mathrm{rand},j}^{i+1}$) and the upper bound corresponding to that coordinate, after being incremented by 1 ($x_{j}^{\max}+1$). Thus, the remainder ($x_{p,\mathrm{rand},j}^{i+1}\%\left(x_{j}^{\max}+1\right)$) varies between 0 and $x_{j}^{\max}$. The mutated coordinate replaces the original coordinate in $x_{p}^{i+1}$ and the result is a new position $x_{p,\mathrm{rand}}^{i+1}$, which has to be non null. If null, all $x_{p,\mathrm{rand}}^{i+1}$ coordinates are generated at random, until the position becomes non null. Like mutation, the randomization aims to enforce climbers explore as much as possible of the search space.

## 3. Results and Discussion

**Figure 5:**
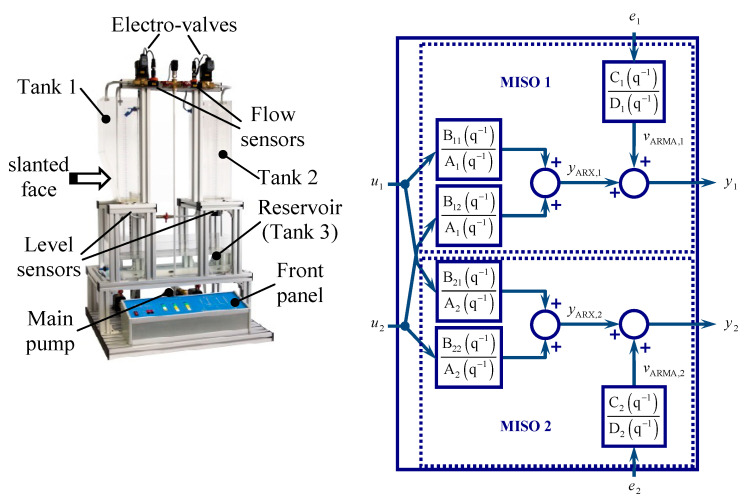
Photo of ASTANK2 installation (**left**) and the scheme of associated multivariable autoregressive identification model (**right**).

The stochastic cubic spline model described in Section 2.1 is tested within a system identification application. The process to identify consists of a two-tank laboratory installation, named *ASTANK2*, which is illustrated on the left side of Figure 5. The upper tanks have distinct constructive characteristics: the left side tank 1 has a slanted face, whereas the right side tank 2 is simply rectangular. The upper tanks are continuously fed with water from the storage reservoir (tank 3), by means of an inverter-driven main pump (which can act in range [0, 10] V) and a flexible (distributed) pipeline network. Thus, the superior tanks are filled by means of a vertical pipe, which is branched in two small horizontal pipes in the upper side. Their inlet flows are controlled through corresponding *electro-valves* (e-v), whose voltage can vary in the same range [0, 10] V. These tanks can be interconnected through a baseline pipe, and, at the same time, evacuated into the inferior tank, with the aid of some manual taps. There are two auxiliary vertical pipes that fill the upper tanks by using two corresponding on-off pumps. The plant is endowed with a set of transducers/sensors (level, flow) and limiters, as well. A detailed description of the installation can be found in [30] or [28]. One focuses next only on the sensors that constitute the main sources of stochastic noises corrupting the acquired data from the installation. 

The ASTANK2 plant is equipped with four static pressure sensors and two volumetric flow sensors. Their characteristics are listed in Table 2. Two of the pressure sensors act as level transducers and are mounted at the bottom of the upper tanks (as Figure 5—left shows). Water level is measured by means of column static pressure. The other two are mounted on the common feed line: one close to the drainage port of the main pump and another one at the top of line (not shown in the figure, as being unimportant). 

The two branches that feed the upper tanks are equipped with volumetric flow sensors. They are mounted nearby the two e-v (see Figure 5—left). The electronic modules they include provide a standard 4–20 mA current outputs, which are accessible as 2–10 V signals.

As Table 2 reveals, while the accuracy of volumetric flow sensors is fair, the static pressure sensors are 10 times more accurate. Nevertheless, in all experiments, the variable of interest is the water level in upper tanks, not the static pressure. Therefore, it is important to correctly calibrate these sensors. The transformation of hydrostatic pressure into water column height is of affine type:

(52) h=a⋅p+b,

where p [bar] is the pressure returned by the sensor, h [cm] is the water height in the tank, whilst a>0 and b≥0 are the two calibration parameters (to be determined experimentally, by means of LSM). To carry out calibration, the procedure is as follows:

1. Empty both tanks.
2. Fully close drainage taps of the tanks and the coupling tap.
3. Fill up both tanks to 5 cm.
4. Record the pressure sensor numeric indication and actual water heights for both tanks.
5. Increase water level in 5 cm increments up to 35 cm and repeat step 4.
6. Use the obtained static h–p characteristics for each tank as experimental data to determine the parameters a>0 and b≥0 (of Equation (52)) by means of LSM.

Since the water level in upper tanks can be affected by turbulences during the filling, the calibration procedure above usually leads to an effective accuracy of ±10%. This produces an observable stochastic noise that corrupts the water height values (as exhibited by all variations of acquired raw data shown in the figures to come). Hence, one can say that those sensors are *noisy*. 

In general, the multitank processes have nonlinear dynamics, governed by the laws of fluid mechanics. Moreover, mainly due to the particular shape of the first tank, the ASTANK2 installation is a nonlinear process. Therefore, the functioning of the plant (without taking into account the presence of the auxiliary pumps), can be modeled by means of a multivariable nonlinear system with three control inputs and two outputs. The inputs are the voltage on the main pump U together with the voltages on the two e-v, namely $u_{1}$ (for the tank 1) and $u_{2}$ (for the tank 2). The outputs are the water levels (heights) in tank 1, $y_{1}$, and tank 2, $y_{2}$, respectively.

Different approaches have been addressed in the modeling/identification of ASTANK2 (analytical model or combination between analytical and experimental models), as reported in [28,30]. In this article, one aims to identify an optimal model of the global installation, starting from available I/O measured data sets. The block scheme of this model is illustrated on the right side of Figure 5 and aims to be integrated in a future automatic control application. In fact, the main purpose here is to prove that the model estimated from the spline (smoothed) output data can be more suitable in control applications (concerning the number of poles exhibited by the useful filter) than the model identified from raw output data.

**Figure 6:**
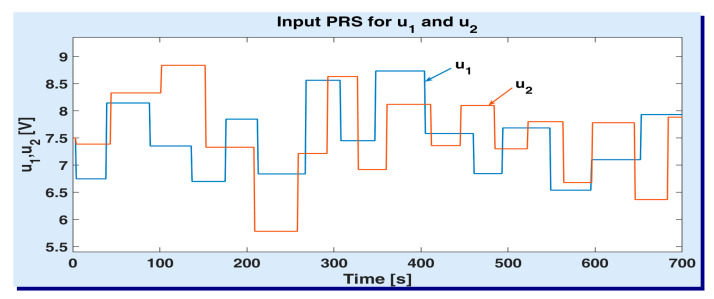
Input signals employed to acquire identification data sets from ASTANK2 installation.

In order to obtain an accurate experimental model, the input signal variations have been bounded by using some physical constraints (experimentally set), which are employed in the usual exploitation of the global plant. In setting such constrains, one wants to avoid: (a) producing turbulences in the upper tanks (especially when the current level of water decreases bellow 2–3 cm); (b) e-v damaging (when the voltage suddenly changes with more than 3 V). More precisely, the stimulating signal U has been maintained at a constant value, whereas each input $u_{1}$, $u_{2}$ has been generated as a normally distributed (Gaussian) *pseudo-random signal* (prs) ranging in the interval [5, 10] V. In addition, the following consistency conditions are enforced: (a) the two input signals ($u_{1}$ and $u_{2}$) are uncorrelated; (b) the duration of a randomly generated voltage value takes from 30 s to 60 s, being generated at random; (c) the variation between two successive values of any input signal is limited to [0.5, 3] V. The minimum and maximum durations of each constant voltage were experimentally set, such that the water inflows produced by the two e-v have time to leave the transient state and enter the steady state. Figure 6 depicts the variations of the input signals $u_{1}$ and $u_{2}$, for the voltage U=9 V of the main pump. The sampling period was set to $T_{s}=1$ s. The illustrated signals were employed to acquire identification data sets on the two output channels. According to Figure 6, the number of output data samples is N=700 for each channel.

**Figure 7:**
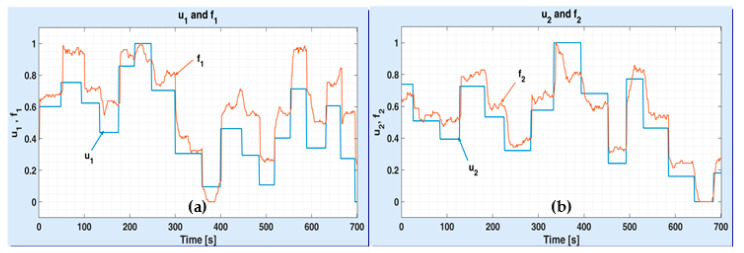
Test input prs to detect transient dynamics of inflows feeding the ASTANK2 main tanks on: channel 1 (**a**) and channel 2 (**b**).

In Figure 7, an example of inflows variations depending on corresponding input prs is given. All variations were normalized in range [0, 1], in order to display voltage-inflow couples on the same window ((a) for channel 1 and (b) for channel 2). The durations of input constant values vary from 20 s to 60 s. The inflows $f_{1}$ and $f_{2}$ succeed to reach for the steady state after 30 s at least and seem to stabilize after 60 s.

As already stated in Section 2.2, two data sets have to be acquired for each output channel: one allocated to model identification and another one employed to validate the identified model (see Equation (37)). Each data set has its relevance in expressing the optimization criteria (38), (41) and (43). The weight λ∈[0,1] can quantify this relevance. In this case study, the two data sets are considered equally relevant. Thus, λ=0.5. The validation data have to be generated by stimulating the plant with different input signals from those employed to generate the identification data, as usually practiced. A very simple way to do it is to switch between the two prs of Figure 6, apply them to the plant inputs and acquire new output data on each channel. According to this technique, overall stimulating inputs have been built by concatenating the identification and validation input signals. Consequently, the output signals $y_{1}$ and $y_{2}$ were acquired during approximately 23 min (1400 samples) with the same sampling period ($T_{s}=1$ s). Obviously, the first 700 samples serve for model identification, whereas the remaining ones are employed in model validation.

**Figure 8:**
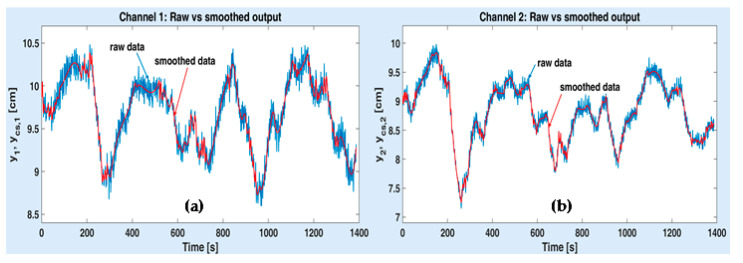
Raw measured and smoothed output signals obtained from channel 1 (**a**) and channel 2 (**b**). of ASTANK2 installation.

For each measured (raw) output signal, the stochastic spline model is estimated (and simulated), by applying the algorithm introduced in Appendix A. This algorithm has been implemented and run within the Matlab programming environment. For each output data set (of 700 samples), the run completed in 0.2–0.4 s, on a regular PC. Figure 8a illustrates the variations of the raw output $y_{1}$ and the corresponding smoothed signal $y_{cs,1}$ for channel 1, on the global measuring horizon. Thus, in the figure, the identification output data are followed by the validation output data (starting at the 701-st second). Similarly, Figure 8b shows the variations of the same signals on channel 2 ($y_{2}$ and $y_{cs,2}$). The noises that affect the raw data are large enough. They are mainly due to the water turbulences that affect the two static pressure sensors (the *noisy* ones), during the data acquisition. 

According to Section 2.2 and Figure 5—right, two MISO models have to be identified (one for each output channel), in order to provide a global model of ASTANK2, as a multivariable process. The identification of each channel relies on the previously described ARX and ARMA models ((27) and (31), respectively). As already mentioned, two optimization strategies have been adopted, in order to find the optimal structure of identification models. Each strategy relies on maximization of SNR-based criteria, namely (38), (41) and (43). Before entering the optimization stage, it is useful to see how such criteria can vary, depending on the structural indices. Because output data are corrupted by stochastic noises (see Figure 8), the (hyper)surfaces that SNR-based criteria can exhibit are expected to have a fractal nature, with many ruptures of first derivative. 

**Figure 9:**
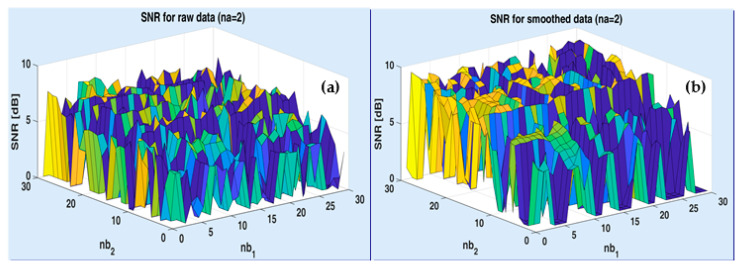
Examples of surfaces exhibited by signal-to-noise ration (SNR)-based criteria for raw output data (**a**) and smoothed data (**b**) on channel 1 of ASTANK2 installation.

For example, Figure 9 illustrates the shapes of two surfaces, corresponding to ARX criterion (38), as estimated from raw and smoothed data, respectively, for channel 1. In this example, na=2, whereas the variable structural indices are $nb_{1}$ and $nb_{2}$. As can be noticed, for the smoothed data (Figure 9), the criterion is slightly less fractal than for the raw data (Figure 9a).

To optimize the three criteria, the AHCA was implemented and run within Matlab programming environment. Thus, nx is either 3 or 2 or 5 (see Equation (45). To limit the search space, the following maximal structural indices were set: for the ARX model, Na=30 and $Nb_{1}=Nb_{2}=50$; for the ARMA model Nc=Nd=100. Since the approximant AR model only plays an auxiliary role, by convention, nα=3max{nc,nd}. This slightly increases the search speed compared to definitions (45). The configuring parameters of AHCA were set to their default values, as shown in the last column of Table 1.

In the sequel, the results obtained after applying the two optimization strategies are compared and discussed.

Usually, in the first (two-step) strategy, for the ARX model, AHCA completes after 3–4 h on a regular PC and the search is terminated when the survival index of the highest climber reaches its maximum value (i.e., 20, according to Table 1). A detail concerning optimization with smoothed data is worth mentioning. Two AHCA runs were initiated. Firstly, one wants to explore the entire search space for an optimal solution na, by setting the upper bound to Na=30 (as mentioned above). After completing the search (even several times), the best na was always found in the $\bar{0,5}$ range. Therefore, secondly, one wants to exploit this narrow variation range, by setting the upper bound to Na=5. In this way, an optimal ARX model with a maximum of five poles is identified. 

Table 3 displays the optimal structural indices of ARX models, as estimated from both raw and smoothed output data for each output channel.

For each channel, one can easily see that na is much smaller when operating with smoothed data than when using the raw (noisy) data (3 versus 30 on channel 1 and 2 versus 27 on channel 2). This result underlines the advantage of exploiting the smoothed data instead of measured ones, in order to estimate an adequate (accurate and parsimonious) useful model of the plant. Clearly, working with smoothed data is more suitable, both in identification and control applications, than using acquired raw data (even after being pre-filtered), especially when the plant is endowed with noisy sensors.

**Figure 10:**
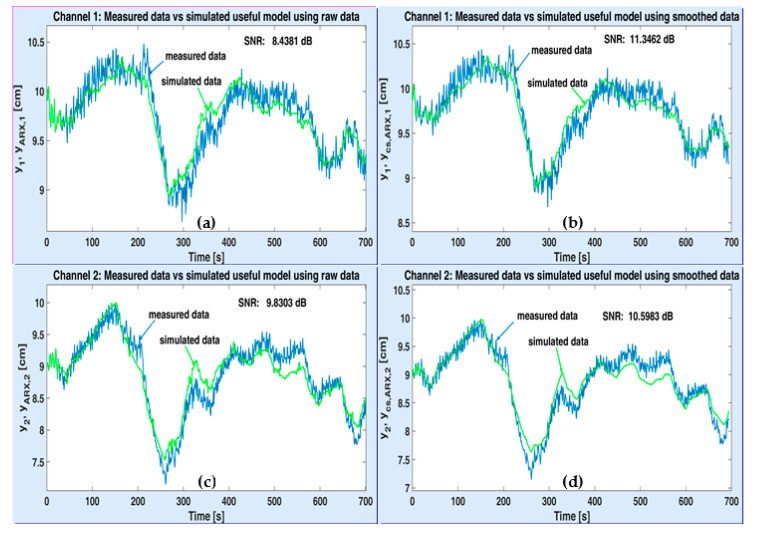
Performance of useful ARX models on channel 1 with raw data (**a**), smoothed data (**b**) and channel 2 with raw data (**c**), smoothed data (**d**).

Figure 10 depicts the variations of measured water levels ($y_{1}$, $y_{2}$) and the simulated useful models ARX, obtained by applying (29), with raw data, ($y_{\mathrm{ARX},1}$, $y_{\mathrm{ARX},2}$) and smoothed data ($y_{cs,\mathrm{ARX},1}$, $y_{cs,\mathrm{ARX},2}$), respectively. All variations are represented on the identification horizon only. Hence, the upper windows ((a) and (b)) refer to channel 1, while the lower windows ((c) and (d)) correspond to channel 2. Moreover, the left side windows ((a) and (c)) deal with raw data, whereas the right side windows ((b) and (d)) are produced by using smoothed data. In each window, the estimated SNR is written. One can notice that, for each channel, the SNR corresponding to smoothed data is slightly higher than the SNR obtained with raw data (11.3462 dB versus 8.4381 dB and 10.5983 dB versus 9.8303 dB). Consequently, the model based on the smoothed data is more accurate, which reinforces the idea of employing smoothed data instead of raw data for estimating the useful component of the plant model.

As mentioned before, the ARMA model is estimated from the residual colored noise, after removing the useful component from the raw measured data. Since the ARX model was obtained from raw/smoothed output data, the ARMA model is estimated accordingly. The corresponding AHCA finds the optimal structure of the ARMA model in approximately 3 h on a regular PC. The optimal structural indices of this model, in case of raw/smoothed data, are revealed in Table 4, for both output channels.

Surprisingly, the colored noise was estimated by simpler optimal models, of *Moving Average* (MA) type (since nd=0), regardless the case. The fact the filter modeling the noise has no poles is remarkable and eases the simulation of plant model.

**Figure 11:**
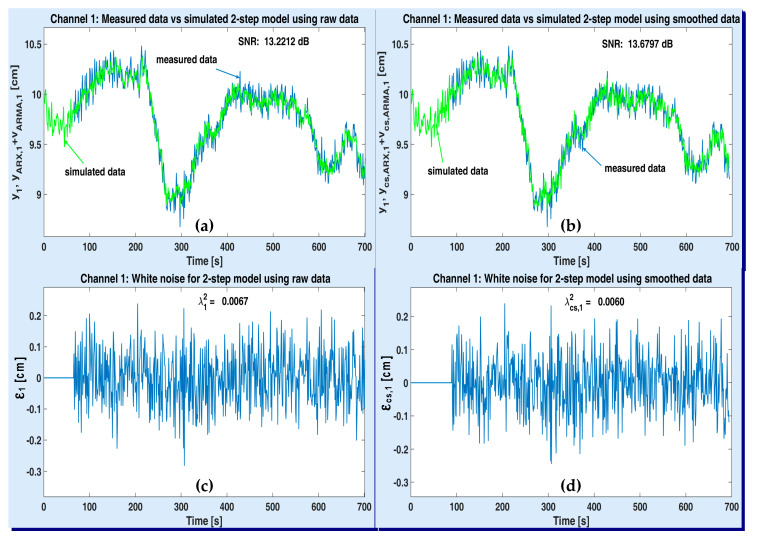
Performance of the optimal 2-step MISO model associated to ASTANK2 installation, on channel 1: measured versus simulated outputs with raw data (**a**) and smoothed data (**b**); estimated white noise from raw data (**c**) and smoothed data (**d**).

**Figure 12:**
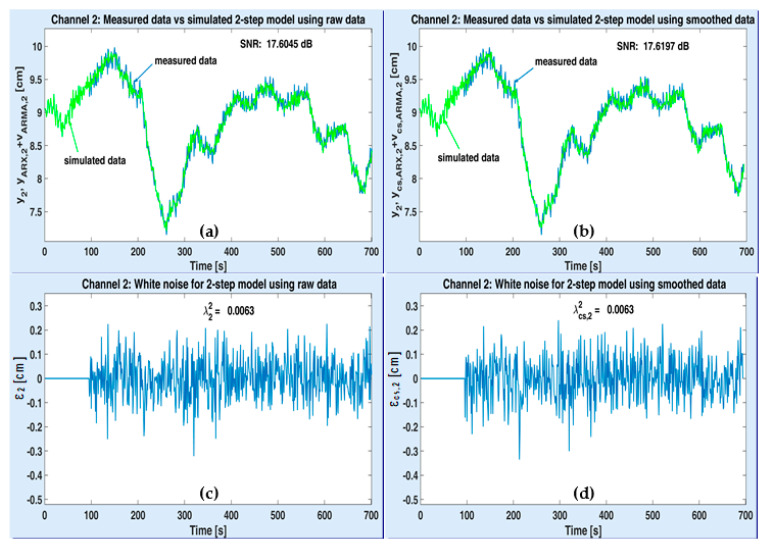
Performance of the optimal 2-step MISO model associated to ASTANK2 installation, on channel 2: measured versus simulated outputs with raw data (**a**) and smoothed data (**b**); estimated white noise from raw data (**c**) and smoothed data (**d**).

Figure 11 and Figure 12 illustrate the simulation results with the optimal MISO models of plant (one for each output channel), after being obtained according to the first strategy (the 2-step one). The variations of the acquired data and the simulated plant model when using raw and smoothed data are depicted on the upper side of both figures (see the windows (a) and (b)). The simulated output of ARMA-AR models was computed by using the recursive system (33). The simulated signals are denoted by $y_{\mathrm{ARX},\{1,2\}}+v_{\mathrm{ARMA},\{1,2\}}$ and $y_{cs,\mathrm{ARX},\{1,2\}}+v_{cs,\mathrm{ARMA},\{1,2\}}$, respectively, according to each output channel $y_{\left\{1,2\right\}}$. The residual white noises were estimated by means of Equation (34). They are shown in the lower part of both figures (see the windows (c) and (d)), together with their corresponding variances, $\lambda_{\left\{1,2\right\}}^{2}$, $\lambda_{cs,\{1,2\}}^{2}$. Since the recursive procedure to compute the simulated useful and noise components has been initialized as explained in Section 2 (i.e., the simulated data are identical to the identification data), the first corresponding values of the residual noises are null.

On upper windows of Figure 11 and Figure 12, the estimated optimal values of corresponding SNR are specified. One can notice that the SNR values obtained with smoothed data are slightly higher than the SNR values estimated with raw data. 

When applying the overall (one-step) optimization strategy, usually the AHCA finds the optimal global model in approximately 5 h on a regular PC. As previously, the ARX model is obtained from raw and smoothed output data. Consequently, two optimal and global MISO models are obtained for each output channel. Table 5 shows their optimal structural indices.

Again, for each channel, the index na is smaller when operating with smoothed data than when using the initial data (1 versus 30 and 2 versus 28), which stresses out the idea of operating with smoothed data for obtaining identification models having a reduced number of poles.

**Figure 13:**
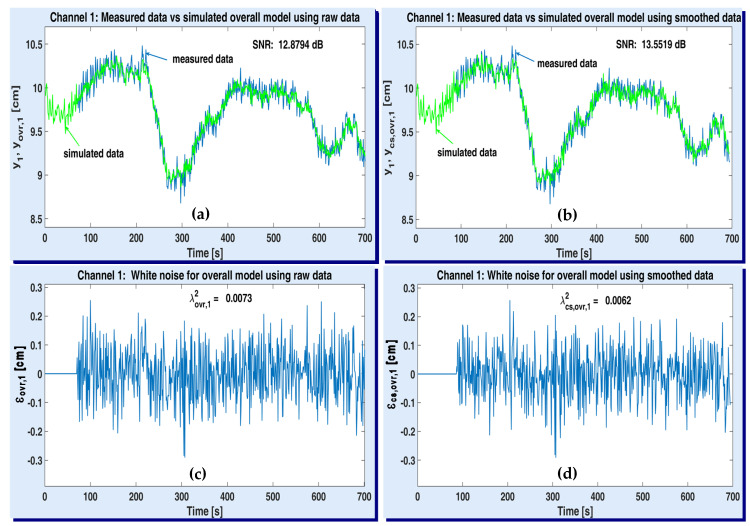
Performance of the optimal overall MISO model associated to ASTANK2 installation, on channel 1: measured versus simulated outputs with raw data (**a**) and smoothed data (**b**); estimated white noise from raw data (**c**) and smoothed data (**d**).

**Figure 14:**
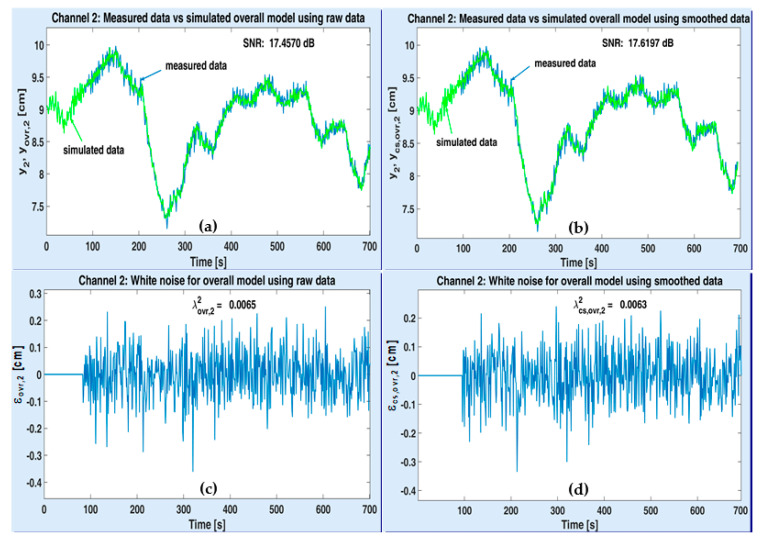
Performance of the optimal overall MISO model associated to ASTANK2 installation, on channel 2: measured versus simulated outputs with raw data (**a**) and smoothed data (**b**); estimated white noise from raw data (**c**) and smoothed data (**d**).

Figure 13 and Figure 14 are similar to Figure 11 and Figure 12, respectively. This time, the performance of the two overall MISO models is analyzed. Therefore, the simulated signals are denoted by $y_{\mathrm{ovr},\{1,2\}}$ and $y_{cs,\mathrm{ovr},\{1,2\}}$, according to each output channel $y_{\left\{1,2\right\}}$. Moreover, the white noises are estimated by means of Equation (36) and their variances are $\lambda_{ovr,\{1,2\}}^{2}$, $\lambda_{cs,ovr,\{1,2\}}^{2}$. In case of overall strategy too, the number of useful poles is reduced by using the smoothed data in identification (1 versus 30 and 2 versus 28). On channel 1, the useful model has one single pole, comparing to three poles as a result of following the previous strategy (see Table 3 and Table 5). On channel 2, the optimal overall model is identical to the optimal 2-step model. Moreover, again, the SNR values of identified models from smoothed data resulted higher than the SNR values of identified models from raw data. Moreover, all optimal noise filters are of MA type (like in one-step strategy), which is remarkable.

Table 6 gathers all SNR values together, as obtained following both strategies.

There is not much difference between the two strategies in terms of estimated SNRs. Nevertheless, AHCA runs faster in the overall strategy (approximately 5 h) than in the 2-step strategy, where two runs have to be initiated: one for ARX and another one for ARMA (all together taking at least 6 h to complete). The main difference is given by the number of useful poles: 1 pole (overall) versus 3 poles (2-step) on channel 1.

## 4. Conclusions

This article aimed to emphasize the advantage of employing the stochastic spline approximation concept in SI, which consists of obtaining a useful model with reduced number of poles. In this respect, an adaptive and optimal stochastic spline model was built, based on LSM with continuity/derivability constraints, so that the provided smoothed data passed as closely as possible to all measured data. The smoothing algorithm is easy to implement and performs fast. The main application is the identification of a real plant (the ASTANK2 installation) starting from two types of data: noisy (directly acquired from the plant through noisy sensors) and smoothed (through stochastic splines). Two optimization strategies in conjunction with an evolutionary algorithm (the AHCA) have been considered, in order to obtain optimal identification models. The simulations have shown that, regardless of the strategy, the optimal models obtained from smoothed data have significantly fewer poles and a slightly better accuracy (higher SNR values) than the optimal models obtained from noisy data. Nevertheless, following the second strategy, the number of useful poles is even slightly smaller than following the first strategy. Models with a reduced number of poles are very welcome in automatic control, since the regulators are, thus, easier to design and implement. In addition, the stochastic spline approximation acts like an adaptive filter for the noisy data. This filter succeeds to perform quite a good separation (though not perfect) between the useful part and the noisy part of raw acquired data. This effect is proven, in subsidiary, by the results obtained after simulations.

## Figures and Tables

**Table 1:** Configuring parameters of AHCA.

Parameter	Meaning	Default Value
*P*	number of climbers in the group	30
*K*	maximum number of altimeter evaluations	30 *P*
*S*	maximum survival factor for the leader	20
*M*	maximum number of mutations per position	20
δ	altitude relative accuracy	5%
*P_e_*	number of elite climbers	3

**Table 2:** Main characteristics of ASTANK2 sensors.

Feature	Static Pressure Sensors	Volumetric Flow Sensors
Type	Siemens SITRANS P210	KOBOLD DPL-1P20
Conversion element	diaphragm with piezo-resistive cell	rotating vane flow meter
Range	0–0.1 bar	0.4–12 L/min
Accuracy	±0.25%	±2.5%
Linearity	±1%	±1%

**Table 3:** Optimal indices for ARX models on both channels in case of two-step optimization strategy.

Channel	Output Data Type	$\mathrm{Optimal}\;\left(na,nb_{1},nb_{2}\right)$
1	Raw	(30,22,16)
1	Smoothed	(**3**,21,47)
2	Raw	(27,7,26)
2	Smoothed	(**2**,2,46)

**Table 4:** Optimal structural indices for ARMA models on both channels, in case of two-step optimization strategy.

Channel	Output Data Type	Optimal (nc,nd)
1	Raw	(65, 0)
1	Smoothed	(89, 0)
2	Raw	(95, 0)
2	Smoothed	(95, 0)

**Table 5:** Optimal structural indices for ARMA models on both channels, in case of one-step optimization strategy.

Channel	Output Data Type	$\mathrm{Optimal}\;\left(na,nb_{1},nb_{2},nc,nd\right)$
1	Raw	(30,23,10,70,0)
1	Smoothed	(**1**,44,16,86,0)
2	Raw	(28,16,11,83,0)
2	Smoothed	(**2**,2,46,95,0)

**Table 6:** Optimal SNR values of MISO models associated to ASTANK2 installation.

Method	Channel	Output Data Type	SNR
2-step	1	Raw	13.2212
2-step	1	Smoothed	**13.6797**
2-step	2	Raw	17.6045
2-step	2	Smoothed	**17.6197**
overall	1	Raw	12.8794
overall	1	Smoothed	**13.5519**
overall	2	Raw	17.4570
overall	2	Smoothed	**17.6197**

## Abbreviation

AHCA	Advanced Hill Climbing Algorithm
ARMA	AutoRegressive with Moving Average (model)
ARMAX	AutoRegressive with Moving Average and eXogenous inputs (model)
ARX	AutoRegressive with eXogenous inputs (model)
HCA	Hill Climbing Algorithm
I/O	Input/Output
LS	Least Squares
LSM	Least Squares Method
MA	Moving Average (model)
MIMO	Multi-Input Multi-Output
MISO	Multi-Input Single-Output
SI	System Identification
SNR	Signal-to-Noise Ratio
dB	deciBells
e-v	electro-valves
prs	pseudo-random signal
std	standard deviation
u-prsg	uniformly distributed pseudo-random sequences generator

## Appendix A

The procedure of building the stochastic cubic spline model is summarized below.

1. Input data:
  ➢ Measured raw data set $D_{N}={\left\{y[n]\right\}}_{n\in \bar{1,N}}$ (here, y is a N-length column vector, in fact);
  ➢ Minimum number of samples in a data subset: $N_{\min}$ (by default, $N_{\min}=10$);
  ➢ Threshold of successive increases realized by std: M (by default, $M=\min\left\{5,N_{\min}\right\}$).
2. Global initialization:
  - Set to void the matrix $\Theta_{cs}$ of subset ending instants and corresponding polynomial coefficients that constitute the cubic spline model (each column of this matrix will have 5 elements: the subset ending instant of “$N_{k}^{e}$” type and the 4 polynomial coefficients);
  - Set to void the simulated spline model, $y_{cs}$ (it will be a column vector).
3. If $N<N_{\min}$, jump to step 6 (end of procedure).
4. Building the first cubic polynomial:
  4.1. Local initialization:
    - Set the previous value of std: $\sigma_{p}=\infty$;
    - Set the initial counter of std increases: m=0;
    - Set $n=N_{\min}$ (the initial length of data subset);
    - Extract the data subset, $y_{dss}=y\left(1:n\right)$, remove the first n values from y and update the data set length, N←N−n.
  4.2. While N≥0 and m<M:
    4.2.1. Identify the first cubic polynomial, corresponding to data subset $y_{dss}$, by using Equation (5). Store the estimated coefficients in $\theta_{cp}^{0}$ (a 4-length column vector).
    4.2.2. Simulate the polynomial by means of Equation (6) (where $M_{1}$ virtually stands for the current length of data subset, i.e., $M_{1}=n$). Store the result in $y_{cs}$.
    4.2.3. Compute the current value of std, $\sigma_{c}$, with $y_{dss}$ and $y_{cs}$, by using definition (26) (where: k=1, $N_{1}^{b}=1$ and $N_{1}^{e}=M_{1}=n$).
    4.2.4. If $\sigma_{c}\leq \sigma_{p}$, reset the counter, m←0.
    4.2.5. Otherwise increment the counter, m←m+1.
    4.2.6. Update the previous value of std, $\sigma_{p}\leftarrow \sigma_{c}$.
    4.2.7. If N>0, extend $y_{dss}$ with the first value in y, increment the subset length, n←n+1 and remove the first value of y.
    4.2.8. Decrement the data set length, N←N−1.
  4.3. If m>0:
    4.3.1. Send back the last m values of $y_{dss}$ to y (in the first positions) and update the data lengths: n←n−m, N←N+m.
    4.3.2. Identify and simulate the cubic polynomial as in steps 4.2.1 and 4.2.2. Store the coefficients in $\theta_{cp}^{0}$ and the simulated data in $y_{cs}$.
  4.4. Compose the first column of matrix $\Theta_{cs}$ by writing the value of n and the vector $\theta_{cp}^{0}$.
5. Building the next cubic polynomials. While $N\geq N_{\min}$:
  5.1. Determine the left side and right side knots, together with their continuity and derivability conditions, according to Equations (17) and (19). In this aim, the last column of matrix $\Theta_{cs}$ and the last value of vector $y_{cs}$ can be employed. If $N=N_{\min}$, then the last 3 data samples (or more) can be employed to compute the average.
  5.2. Perform local initialization as in step 4.1.
  5.3. While N≥0 and m<M:
    5.3.1. Identify the cubic polynomial corresponding to data subset $y_{dss}$, with the help of Equations (14) and (11), i.e., by only considering continuity conditions (9). Store the estimated coefficients in $\theta_{cp}^{0}$.
    5.3.2. Simulate the polynomial by means of Equation (8) and $\theta_{cp}^{0}$ above (where $N_{k}^{b}$ is obtained after incrementing by 1 the first element of last column in $\Theta_{cs}$, whilst $N_{k}^{e}=N_{k}^{b}+n-1$). Store the result in $y_{cp}^{0}$ (column vector).
    5.3.3. Compute the current value of std, $\sigma_{c}^{0}$, with $y_{dss}$ and $y_{cp}^{0}$, by using definition (26) (where $N_{k}^{b}$ and $N_{k}^{e}$ are obtained as in step 5.3.2).
    5.3.4. Identify the cubic polynomial corresponding to data subset $y_{dss}$, with the help of Equations (25) and (21), i.e., by considering the full set of conditions (19). Store the estimated coefficients in $\theta_{cp}^{1}$ (a 4-length column vector).
    5.3.5. Simulate the polynomial by means of Equation (8) and $\theta_{cp}^{1}$ above (where $N_{k}^{b}$ and $N_{k}^{e}$ are obtained as in step 5.3.2). Store the result in $y_{cp}^{1}$ (column vector).
    5.3.6. Compute the current value of std, $\sigma_{c}^{1}$, with $y_{dss}$ and $y_{cp}^{1}$, by using definition (26) (where $N_{k}^{b}$ and $N_{k}^{e}$ are obtained as in step 5.3.2).
    5.3.7. Compute the minimum value of std: $\sigma_{c}=\min\left\{\sigma_{c}^{0},\sigma_{c}^{1}\right\}$.
    5.3.8. If $\sigma_{c}\leq \sigma_{p}$, reset the counter, m←0.
    5.3.9. Otherwise increment the counter, m←m+1.
    5.3.10. Update the previous value of std, $\sigma_{p}\leftarrow \sigma_{c}$.
    5.3.11. If N>0, extend $y_{dss}$ with the first value in y, increment the subset length, n←n+1 and remove the first value of y.
    5.3.12. Decrement the data set length, N←N−1.
  5.4. If m>0:
    5.4.1. Send back the last m values of $y_{dss}$ to y (in the first positions) and update the data lengths: n←n−m, N←N+m.
    5.4.2. Identify and simulate the two cubic polynomials as in steps 5.3.1, 5.3.2, 5.3.4, 5.3.5. Store the coefficients and the simulated data in $\theta_{cp}^{0}$, $\theta_{cp}^{1}$, $y_{cp}^{0}$, $y_{cp}^{1}$, respectively.
    5.4.3. Compute the two stds as in steps 5.3.3 and 5.3.6. Store their values in $\sigma_{c}^{0}$ and $\sigma_{c}^{1}$, respectively.
  5.5. If $\sigma_{c}^{0}>\sigma_{c}^{1}$, then the first polynomial is a better fit for the data subset than the second one. Set b=0 (the flag of best polynomial).
  5.6. Otherwise, the second polynomial is the best among the two. Set b=1.
  5.7. Final operations: extend $y_{cs}$ by $y_{cp}^{b}$ and $\Theta_{cs}$ by a column that includes the subset ending instant and the estimated parameters vector $\theta_{cp}^{b}$. The new ending instant is obtained by adding the current subset length, n, to the previous ending instant (the first element of last column in $\Theta_{cs}$, before extension).
6. Return $y_{cs}$ and $\Theta_{cs}$.

Some remarks are useful, concerning the procedure above:

- As the step 4 suggests, it is possible to ignore the last $N_{\min}-1$ (or less) raw data. If this is unacceptable, the few remaining data (in number of $N_{\min}-1$, at most) can be added to the last subset. In this case, the corresponding cubic polynomial has to be identified and simulated again. To do so, the right side knot can be created by averaging the last 3 data (or more).
- The final cubic polynomial is not necessarily the one with minimum std. If the minimum std value is employed to select the optimal polynomial, simulations have shown that, in general, the adaptively selected data subsets are too short and the spline curve is oscillating too much, trying to get closer to the raw data. This means an important amount of noise is transferred to the spline model, which leads to a very poor data smoothing effect.
- Using two polynomials instead of a single one yields smoothing a large class of raw data. If the spline model is continuous but not necessarily derivable in all knots, then the final fitting curve could look quite fractal, as if the noise was corrupting not only the data, but the model too. If the spline model has to be derivable in all knots, then data with sudden changes in their average could lead to visible biases of fitting curve, comparing to the data. For example, if a delayed step is sunk into a white noise, the fully derivable cubic spline could poorly approximate the sudden change in data. By adaptively selecting which piecewise polynomial (among the two ones) is the fittest, the smoothing effect is quite good.
